# Next-generation sequencing applied to a large French cone and cone-rod dystrophy cohort: mutation spectrum and new genotype-phenotype correlation

**DOI:** 10.1186/s13023-015-0300-3

**Published:** 2015-06-24

**Authors:** Elise Boulanger-Scemama, Said El Shamieh, Vanessa Démontant, Christel Condroyer, Aline Antonio, Christelle Michiels, Fiona Boyard, Jean-Paul Saraiva, Mélanie Letexier, Eric Souied, Saddek Mohand-Saïd, José-Alain Sahel, Christina Zeitz, Isabelle Audo

**Affiliations:** INSERM, U968, Paris, F-75012 France; Institut de la Vision, Sorbonne Universités, UPMC Univ Paris 06, UMR_S 968, 17, rue Moreau, Paris, F-75012 France; CNRS, UMR_7210, Paris, F-75012 France; IntegraGen SA, Genopole CAMPUS 1 bat G8 FR-91030 EVRY, Paris, France; Centre Hospitalier Intercommunal de Créteil, Department of Ophthalmology, Université Paris-Est Créteil, 94000 Créteil, France; Centre Hospitalier National d’Ophtalmologie des Quinze-Vingts, DHU ViewMaintain, INSERM-DHOS CIC 1423, Paris, F-75012 France; Fondation Ophtalmologique Adolphe de Rothschild, 75019 Paris, France; Académie des Sciences-Institut de France, 75006 Paris, France; University College London Institute of Ophthalmology, 11-43 Bath Street, London, EC1V 9EL UK

**Keywords:** Inherited retinal disorders, Cone-rod dystrophy, Next-generation sequencing, Genotype-phenotype correlation

## Abstract

**Background:**

Cone and cone-rod dystrophies are clinically and genetically heterogeneous inherited retinal disorders with predominant cone impairment. They should be distinguished from the more common group of rod-cone dystrophies (retinitis pigmentosa) due to their more severe visual prognosis with early central vision loss. The purpose of our study was to document mutation spectrum of a large French cohort of cone and cone-rod dystrophies.

**Methods:**

We applied Next-Generation Sequencing targeting a panel of 123 genes implicated in retinal diseases to 96 patients. A systematic filtering approach was used to identify likely disease causing variants, subsequently confirmed by Sanger sequencing and co-segregation analysis when possible.

**Results:**

Overall, the likely causative mutations were detected in 62.1 % of cases, revealing 33 known and 35 novel mutations. This rate was higher for autosomal dominant (100 %) than autosomal recessive cases (53.8 %). Mutations in *ABCA4* and *GUCY2D* were responsible for 19.2 % and 29.4 % of resolved cases with recessive and dominant inheritance, respectively. Furthermore, unexpected genotype-phenotype correlations were identified, confirming the complexity of inherited retinal disorders with phenotypic overlap between cone-rod dystrophies and other retinal diseases.

**Conclusions:**

In summary, this time-efficient approach allowed mutation detection in the most important cohort of cone-rod dystrophies investigated so far covering the largest number of genes. Association of known gene defects with novel phenotypes and mode of inheritance were established.

**Electronic supplementary material:**

The online version of this article (doi:10.1186/s13023-015-0300-3) contains supplementary material, which is available to authorized users.

## Background

Cone and cone-rod dystrophy (CCRD) refer to a heterogeneous group of inherited retinal disorders (IRDs), characterized by predominant cone impairment. They are the most common cause of hereditary cone dysfunction, with a prevalence of 1:40000 [1]. Patients typically complain of progressive central visual loss associated with photophobia and colour vision abnormalities in childhood or early adult life. In case of associated rod system involvement, patients may also experience dim light vision disturbances and peripheral visual field constriction, leading to severe visual loss and complete blindness in some cases [1]. On fundoscopy, the macular appearance ranges from normal to bull’s eye maculopathy or more severe macular atrophy with possible pigmentary changes in the periphery in case of rod photoreceptor involvement [2]. Full-field electroretinogram (ERG) is the key examination for diagnosis and reveals predominant cone dysfunction with rod responses initially normal or minimally impaired. Advanced stages are characterized by both cone and rod impairment making the differential diagnosis with rod-cone dystrophy (or Retinitis Pigmentosa, RP) difficult. Progressive CCRD need to be distinguished from cone dysfunction syndromes, which are typically stationary, congenital with normal rod function [3]. However, these two entities have some phenotypic overlaps with difficulties for differential diagnosis when the congenital onset is not clearly documented. In addition CCRD often presents as an isolated disease, but can also be part of a syndrome as in Bardet-Biedl, Jalili syndrome or Spinocerebellar ataxia 7 [1, 5–7].

The genetic basis of CCRD is highly heterogeneous. Inheritance of CCRD can be either autosomal recessive (ar), autosomal dominant (ad) or X-linked (xl). Simplex CCRD are also frequent for which inheritance pattern is difficult to determine. A recent review estimated that ar (including isolated cases), ad and xl inheritance were found in 77 %, 22 %, and 1 % of CCRD, respectively [8]. To date, mutations in 30 genes have been implicated in CCRD (https://sph.uth.edu/retnet/ March 2015). Mutations in *ABCA4* (ATP-binding cassette, sub-family A, member 4) [8, 9], *GUCY2D* (Guanylate Cyclase 2D) [10, 11] and *RPGR* (Retinitis Pigmentosa GTPase regulator) [12, 13] are major causes of ar, ad, and xl CCRD respectively. Novel gene defects still need to be identified since recent comprehensive studies genetically resolved less than 25 % of ar CCRD [2, 8]. Furthermore, clinical and genetic overlaps exist between CCRD and other IRDs. Distinct mutations in a same gene can cause distinct phenotypes, thereby leading to new phenotype-genotype correlations. For example, mutations in *ABCA4, CRX, CERKL, PROM1, SEMA4A, GUCY2D* can cause either CCRD, but also RP or Leber congenital amaurosis (LCA). In this context, Next Generation Sequencing (NGS) targeting not only genes known to underlie CCRD but also more comprehensively other genes mutated in IRDs provides the method of choice, compared to Sanger sequencing, to encompass clinical and genetic heterogeneity of this disease group [14]. Targeted NGS has been successfully applied for investigating IRD: studies covering from 45 to 254 known genes implicated in IRDs were able to genetically resolve from 25 % to 57 % of cases [14–20]. In contrast, only one study so far applied NGS, targeting 25 genes, to CRD [21]. The purpose of our study was to conduct a more comprehensive analysis of CCRD by applying a NGS panel covering 123 genes, improved from a previous report [14], to a French cohort of 96 clinically well characterized patients (95 index patients) who had never been genetically investigated and therefore assess the distribution and prevalence of mutations and genes involved in CCRD.

## Methods

### Clinical diagnosis of CCRD

Ninety-six patients, from 95 unrelated families (2 siblings) with a presumed diagnosis of non-syndromic CCRD, were included. Inheritance was determined considering the transmission pattern of the disease phenotype in the family. Ad transmission was clearly established for 13 patients (1 of the 2 parents affected, transmission to offspring, male and female affected with equal frequency, male-to-male transmission). Ar transmission was strongly suspected when consanguinity was present in the family or when only one generation was affected by the disease (39 patients). The remaining 44 patients were isolated CCRD for whom no clear inheritance pattern could be determined (sporadic cases). Consanguinity was present in 31 families. NGS does not capture the repetitive region ORF15 of *RPGR*, implicated in X-linked CCRD, which is a limit of the NGS technique. To avoid this hurdle, we voluntarily excluded patients with suspected x-linked transmission, by including only women (*n* = 76), or men with father-son transmission (*n* = 2) or reported consanguinity (*n* = 18) in parents. Thus in theory patients with x-linked transmission were excluded from this studied population. This should however be taken with caution since in RP, some female carriers of mutated x-linked genes can also reveal a disease phenotype [22]. Each patient underwent full ophthalmic examination as described earlier [23]. Total genomic DNA was extracted from peripheral blood samples according to manufacturer recommendations (Puregen Kit, Qiagen, Courtabœuf, France). Written informed consent was obtained from each patient after explanation of the study and its potential outcomes. The study protocol adhered to the tenets of the Declaration of Helsinki and was approved by the regional ethical committee (CPP, *Comité de Protection des Personnes* Ile de France V).

### Molecular genetic analysis using NGS

A custom-made SureSelect oligonucleotide probe library was previously designed in collaboration with a company (IntegraGen, Evry, France) to capture the exons of 254 known or candidate genes underlying retinal disorders (Retinal Information Network Database https://sph.uth.edu/retnet/, Pubmed database http://www.ncbi.nlm.nih.gov/pubmed/, October 2010) [14]. The panel was subsequently improved for better coverage and cost efficiency. We first excluded candidate genes for inherited retinal diseases present in the initial panel, now that the candidate genes strategy has been replaced by Whole Exome Sequencing for unsolved patients after targeted NGS. In addition, we also chose to exclude genes that are associated with a pathognomonic phenotype when mutated that can be directly screened by Sanger technique for cost efficiency. These include *CNGA3, CNGB3* and *PDE6H* which are associated with congenital cone dysfunction syndrome (i.e. achromatopsia) that may be associated with mild disease progression but is distinct from cone dystrophy which is not congenital [24]. Similarly, *KCNV2* and *CACNA1F* when mutated lead to distinct electrophysiological phenotypes (namely the so-called super normal rod ERG and a Schubert-Bornschein type of ERG respectively) [25, 26]. *CNNM4* was also excluded from the list of targeted genes since mutations in this gene are distinctively associated with amelogenesis imperfecta as part of Jalili syndrome [27]. In addition, the implication of three novel genes *C21Orf2* [28]*, RAB28* [29] and *TTLL5* [30] in CCRD were identified and published after the design of the panel and consequently were not included in this study. The panel was therefore reduced from 254 to 123 genes implicated in IRDs including 21 CCRD causative genes and 102 other IRD genes (https://sph.uth.edu/retnet/, March 2015, Additional file 1).

Sequence capture, enrichment, and elution were performed according to Agilent’s instruction. Subsequent genomic alignment (with UCSC hg19 for the reference) and sequence variation annotations were performed as previously described [14].

### Filtering approach

In order to identify disease-causing mutations among non-pathogenic single nucleotide variants, we selected exonic non-synonymous variants and splice site variants (±5 base pair apart from exon). Subsequently, we applied a systematic filter based on the known pattern of inheritance of the gene defect (heterozygous variant in ad, compound heterozygous or homozygous in ar) and the allelic frequency reported in public database (dbSNP, HapMap, 1000 Genome, Exome Variant Server) to be ≤ 0.5 % for ar and to be ≤ 0.1 % for ad cases.

### Pathogenic prediction of variants

Frameshift, nonsense, splicing mutations were considered as most likely disease causing. For missense mutations, amino acid conservation was studied across 50 species in UCSC Genome Browser (http://genome.ucsc.edu/). The amino acid was considered as « highly conserved » if the residue did not change. Between 1 and 5 variations (different from the mutation and not in primates), it was considered as « moderately conserved ». Between 5 and 7 variations, it was considered as « weakly conserved ». Otherwise the amino acid was considered as « not conserved ». Pathogenicity of missense was also evaluated on the basis of bioinformatic predictions as Polyphen (Polymorphism Phenotyping, http://genetics.bwh.harvard.edu/pph2/) [31], SIFT (Sorting Intolerant From Tolerant, http://sift.jcvi.org/www/SIFT_enst_submit.html) [32] and Mutation Taster [33]. We considered the amino acid exchange as pathogenic, when it was predicted to be disease causing by at least one of the programs.

To identify previously reported pathogenic variants, literature was systematically reviewed for each selected variant (The Human Gene Mutation Database (HGMD) Pro, Leiden Open Variation Database (LOVD), Retina International, Ensembl, NCBI Pubmed).

### Sanger sequencing validation and co-segregation analysis

All most likely pathogenic variants were confirmed with direct PCR and Sanger sequencing. Pair of oligonucleotides were manually designed at least 50 bp upstream and downstream from the mutation for PCR-amplification and sequencing. Amplicons were enzymatically purified (ExoSAP-IT, USB Corporation, Cleveland, Ohio, USA purchased from GE Healthcare, Orsay, France) and sequenced with a commercially available sequencing mix (BigDyeTerm v1.1 CycleSeq kit, Applied Biosystems, Courtabœuf, France). The sequenced products were purified on a pre-soaked Sephadex G-50 (GE Healthcare) 96-well multiscreen filter plate (Millipore, Molsheim, France), the purified product analyzed on an automated 48-capillary sequencer (ABI 3730 Genetic analyzer, Applied Biosystems) and the results interpreted by applying a software (SeqScape, Applied Biosystems). The nomenclature of each variant was checked applying Mutalyzer (https://mutalyzer.nl/check) to concord with the Human Variation Genome Society guidelines for mutation nomenclature (http://www.hgvs.org/mutnomen/).

When blood samples from family members were available, co-segregation analysis was performed to confirm association of selected variants with the phenotype.

### Multiplex ligation-dependent probe amplification (MLPA) analysis for *ABCA4*

Patients found to carry only one pathogenic variant *ABCA4* as well as patients with no pathogenic variant after targeted NGS analysis were investigated for putative large deletions by a kit (SALSA MLPA probemix P151-B1/P152-B2 ABCA4, MRC-Holland, Amsterdam, Netherland).

### Copy number variations (CNVs) analysis

To detect large deletion or duplication in cases where targeted NGS did not reveal a disease causing variant or only one heterozygous variant in recessive cases, we developed an algorithm to extract CNVs from exome depth obtained from NGS raw data after genomic alignment (BAM files). All depth files from a set of samples to be analyzed (test set) were compiled and correlated with files from a depth reference set obtained from the same sequencing tool with the same NGS strategy. Validation for comparison was made if the correlation coefficient was >0.97. Individual depth was then compared to the depth of the reference set for each sample and each target and a score was generated based on the presumed number of copies within the targeted region. Targets with a score ≤ 0.5 (suspected of deletion) or ≥1.5 (suspected of duplication) were selected and subsequently confronted to data from the general population reported in CNV databases (e.g. Database of Genomic Variants, http://dgv.tcag.ca/dgv/app/about) to exclude common variants (>0.5 % for recessive cases and >0.1 % for dominant cases). Putative pathogenic CNVs were checked by direct Sanger sequencing (*MERTK*), MLPA (reagent from MRC-Holland SALSA MLPA probemix P367-A1 BEST1-PRPH2 kit, for *PRPH2*) and by qPCR for *FSCN2* exon 1 deletion [34].

## Results

### NGS capture and coverage

**Fig. 1:**
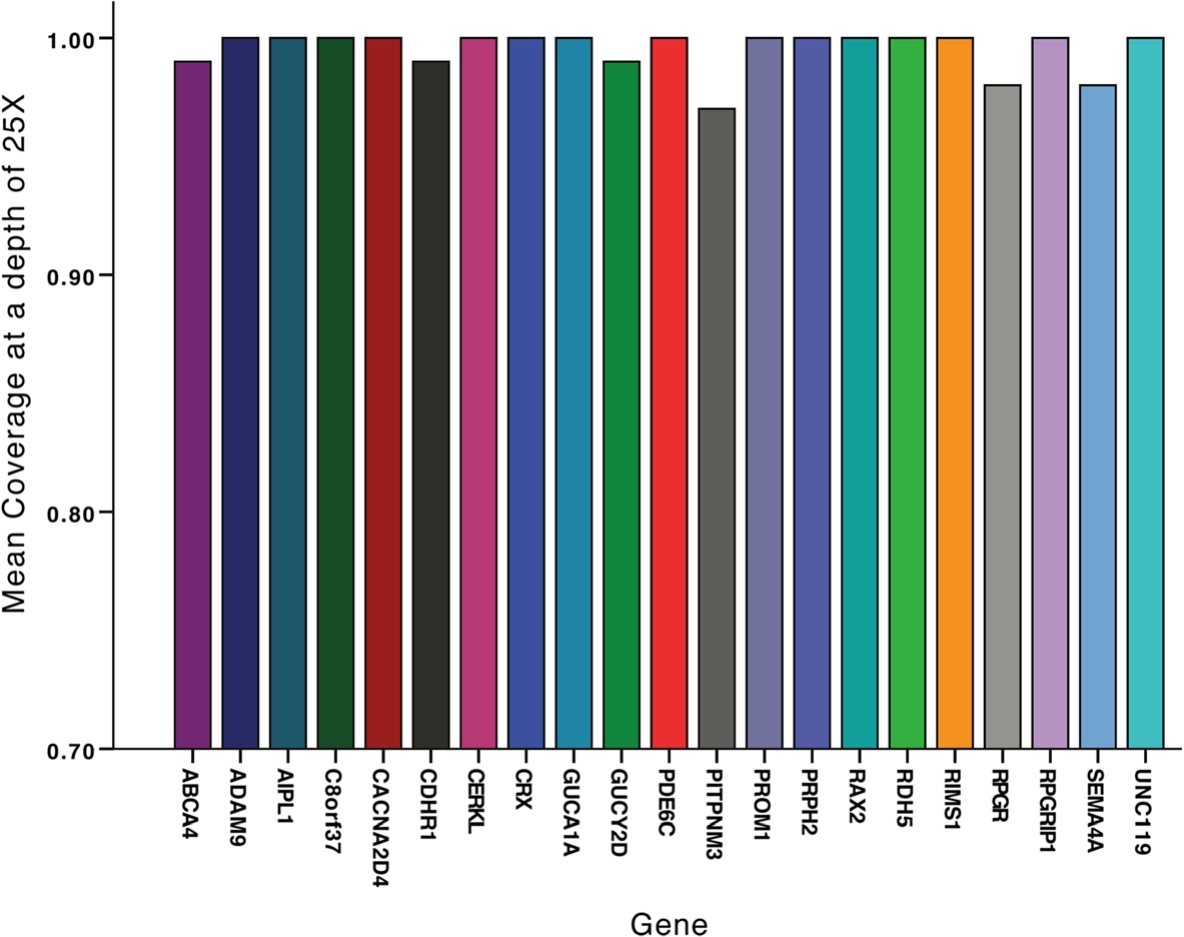
Percentage of regions with 25-fold coverage per base for each of the 21 known CCRD genes of the panel. The mean and median coverage per gene were 244-fold and 248-fold per base, respectively, with a minimal coverage of 171-fold for *RAX2*

For most of the 21 known CCRD genes of the panel, 100 % of the targeted regions had at least 25-fold coverage per base beside *ABCA4*, *CDHR1*, and *GUCY2D* which were at least 99 % covered at 25X whereas *PITPM3* was covered at 97 %, *RPGR* at 98 % and *SEMA4A* at 98 % (Fig. 1). The mean and median coverage per gene were 244-fold and 248-fold per base, respectively, with a minimal coverage of 171-fold for *RAX2*. For some regions, coverage was low (<25-fold per base) or null in several to almost all samples, in relation with GC-rich sequences (*IMPDH1* exon 1 (95/96 samples), *PEX7* exon 1 (87/96 samples), *PITPNM3* exon 1 (35/96 samples), *FLVCR1* exon 1 (2/96 samples), *RP9* exon 1 (51/96) and *CHM* exon 5). NGS is therefore not a good method for these particular regions which were studied by Sanger sequencing for patients with no pathogenic variants after NGS analysis.

On average 450 Single Nucleotide Variants (SNVs) and 57 insertions/deletions were identified per sample.

### Identification of pathogenic mutations

*Pathogenic mutations in known CCRD genes* As listed in Table 1, a total of 49 different pathogenic or likely pathogenic variants were found in 11 known CCRD genes, including 22 previously reported mutations, 17 novel loss-of-function (deletion, frameshift, nonsense, splice site variants) and 10 novel missense mutations. These variants were identified in 42 patients (21 sporadic cases, 11 ar and 10 ad cases). Interestingly for 2 patients initially considered as sporadic cases, NGS data led to the identification of heterozygous mutations in genes implicated in ad CCRD. Further clinical investigation of the patients’ family members revealed that these patients had finally dominant inheritance (CIC06757, *PRPH2* het c.514C>T p.(R172W) and CIC05563, *SEMA4A* het c.302T>C p.(Ile101Thr)) (Additional file 2).

**Table 1:** Summary of 43 patients carrying pathogenic and likely pathogenic mutations in known CCRD genes

ID	Type	Consang.	Gene	NM	Allele State	Exon	cDNA	Protein	Coseg.	Conservation	Polyphen2	Sift	Mutation taster	References
*High confidence*														
CIC00137	simplex		*ABCA4*	NM_000350.2	Ho	47	c.6394G>A	p.(E2132K)	+	Highly	Prd	D	Dc	Novel
CIC00765	Ar	+	*ABCA4*	NM_000350.2	Ho	47	c.6445C>T	p.(R2149*)	+	-	-	-	-	(Lewis et al. 1999) (rs61750654)
CIC03436	Ar	+	*ABCA4*	NM_000350.2	Ho	42	c.5892del	p.(G1965Efs*9)	Np	-	-	-	-	^a^
CIC04412	simplex		*ABC4A*	NM_000350.2	Het	34	c.4793C>A	p.(A1598D)	+	Weakly	Pd	T	Dc	(rs61750155) [9]
			*ABCA4*	NM_000350.2	Het	28	c.4234C>T	p.(Q1412*)	+	-	-	-	-	(rs61750137) [9]
CIC04645	Ar	+	*ABCA4*	NM_000350.2	Ho	13	c.1924T>C	p.(F642L)	Np	Moderately	B	D	Dc	Novel, but c.1924T>A p.F642I in [80]
CIC05087	simplex		*ABCA4*	NM_000350.2	Ho	IVS 11	c.1554+1G>C	r.(spl?)	Np	Highly	-	-	-	Novel
CIC05853	simplex	+	*ABC4A*	NM_000350.2	Ho	22	c.3259G>A	p.E1087K	Np	Highly	Prd	D	Dc	(rs61751398) [81]
CIC05854	Ar	+	*ABC4A*	NM_000350.2	Ho	35	c.4919G>A	p.(R1640Q)	+	Highly	Prd	D	Dc	(rs61751403) [82]
CIC05989	simplex		*ABC4A*	NM_000350.2	Het	34	c.4837G>A	p.(D1613N)	+	Not	B	D	Dc	Novel
			*ABC4A*	NM_000350.2	Het	10	c.1302del	p.(Q437Rfs*12)	+	-	-	-	-	Novel
			*ABCA4*	NM_000350.2	Het	38	c.5318C>T	p.(A1773V)	+	Moderately	Prd	D	Dc	[83]
CIC06170	simplex		*ABC4A*	NM_000350.2	Het	44	c.6089G>A	p.(R2030Q)	+	Highly	Prd	D	Dc	(Lewis et al. 1999) (rs61750641)
			*ABC4A*	NM_000350.2	Het	IVS 24	c.3607+3A>T	r.(spl?)	+	Moderately	-	-	-	Novel
			*ABCA4*	NM_000350.2	Het	14	c.2034G>T	p.(K678N)	+	Highly	Prd	D	Dc	[84]
CIC06735	Ar	+	*ABC4A*	NM_000350.2	Ho	42	c.5892del	p.(G1965Efs*9)	Np	-	-	-	-	^a^
CIC06913	Ar	+	*ABCA4*	NM_000350.2	Ho	21	c.3056C>T	p.(T1019M)	+	Highly	Prd	D	Dc	(rs201855602) [85]
CIC04239	Ar	+	*CDHR1*	NM_033100.3	Ho	9	c.838C>T	p.(R280*)	Np	-	-	-		Novel
CIC06568	Ar	+	*CERKL*	NM_001030311.2	Ho	8	c.1090C>T	p.(R364*)	Np	-	-	-	-	Thesis (Sergouniotis P. 2012) ^b^
CIC07299	simplex	+	*PDE6C*	NM_006204.3	Ho	2	c.542del	p.(A181Efs*13)	Np	-	-	-	-	Novel
CIC05218	Ar	+	*PDE6C*	NM_006204.3	Ho	IVS 10	c.1413+3A>T	r.(spl?)	Np	Not	-	-	-	Novel
CIC05563	Ad		*SEMA4A*	NM_022367.3	Het	4	c.302T>C	p.(I101T)	+	Moderately	Prd	D	Dc	Novel (rs149652495)
CIC07563	simplex		*SEMA4A*	NM_022367.3	Ho	3	c.241C>T	p.(R81*)	Np	-	-	-	-	Novel
CIC00324	Ad		*GUCY2D*	NM_000180.3	Het	13	c.2512C>T	p.(R838C)	+	Highly	Prd	D	Dc	(rs61750172) [10]
CIC03249	Ad		*GUCY2D*	NM_000180.3	Het	13	c.2512C>T	p.(R838C)	+	Highly	Prd	D	Dc	(rs61750172) [10]
CIC04347	Ad		*GUCY2D*	NM_000180.3	Het	13	c.2512C>T	p.(R838C)	+	Highly	Prd	D	Dc	(rs61750172) [10]
CIC04918	Ad		*GUCY2D*	NM_000180.3	Het	13	c.2512C>T	p.(R838C)	Np	Highly	Prd	D	Dc	(rs61750172) [10]
CIC06757	Ad		*PRPH2*	NM_000322.4	Het	1	c.514C>T	p.(R172W)	+	Moderately	Prd	D	Dc	(rs61755792) [86]
CIC03621	Ad		*PRPH2*	NM_000322.4	Het	1	c.1-c581+?del	-	+	-	-	-	-	Novel
CIC00535	Ad		*PROM1*	NM_006017.2	Het	10	c.1117C>T	p.(R373C)	+	Not	Pd	D	Dc	(rs137853006) [3]
CIC01196	simplex		*PROM1*	NM_006017.2	Ho	12	c.1354dup	p.(Y452Lfs*13)	+	-	-	-	-	[71]
CIC07045	simplex		*PROM1*	NM_006017.2	Ho	IVS 17	c.1984-1G>T	r.(spl?)	Np	Highly	-	-	-	Novel (rs373680665)
CIC06642^c^	Ad		*PROM1*	NM_006017.2	Het	1	c.7dup	p.(L3Pfs*28)	+	-	-	-	-	Novel
CIC06698^c^	Ad		*PROM1*	NM_006017.2	Het	1	c.7dup	p.(L3Pfs*28)	+	-	-	-	-	Novel
CIC04945	simplex		*PROM1*	NM_006017.2	Het	23	c.2383T>C	p.(W795R)	+	Highly	Prd	D	Dc	Novel
			*PROM1*	NM_006017.2	Het	IVS 13	c.1579-1G>C	r.(spl?)	+	Highly	-	-	-	Novel
CIC04965	Ad		*CRX*	NM_000554.4	Het	4	c.608_609del	p.(S203Ffs*32)	+	-	-	-	-	Novel
CIC3750	simplex		*CRX*	NM_000554.4	Het	3	c.121C>T	p.(R41W)	+	Highly	Prd	D	Dc	(rs104894672) [70]
CIC06321	simplex	+	*RPGRIP1*	NM_020366.3	Ho	14	c.2021C>A	p.(P674H)	+	Highly	Prd	T	Dc	Novel
CIC00190	simplex		*AIPL1*	NM_014336.4	Het	5	c.769C>T	p.(L257F)	+	Moderately	Prd	D	Dc	Novel
			*AIPL1*	NM_014336.4	Het	5	c.767T>G	p.(I256S)	+	Moderately	B	D	Dc	Novel
*Lower confidence* ^d^														
CIC00162	Ar		*ABCA4*	NM_000350.2	Het	31	c.4546_4547del	p.(Q1516Afs*38)	+	-	-	-	-	Novel
			*ABCA4*	NM_000350.2	Het	16	c.2463G>A	p.(W821*)	+	-	-	-	-	Novel
CIC05987	Ar		*ABC4A*	NM_000350.2	Het	22	c.3295T>C	p.(S1099P)	+	Highly	Pd	D	Dc	(rs61750119) [87]
			*ABC4A*	NM_000350.2	Het	22	c.3322C>T	p.(R1108C)	Np	-	-	-	-	[88]
CIC06694	simplex		*ABC4A*	NM_000350.2	Het	IVS36	c.5196+1G>A	r.(spl?)	Np	-	-	-	-	[62]
			*ABC4A*	NM_000350.2	Het	22	c.3322C>T	p.(R1108C)	Np	-	-	-	-	[86]
CIC02712	simplex	+	*PDE6C*	NM_006204.3	Het	10	c.1325T>A	p.(M442K)	Np	Moderately	Pd	D	Dc	Novel
			*PDE6C*	NM_006204.3	Het	10	c.1375C>G	p.(Q459E)		Weakly	B	T	Dc	Novel
CIC00597	simplex		*GUCY2D*	NM_000180.3	Het	14	c.2747T>C	p.(I916T)	+	Moderately	Prd	D	Dc	[89]
CIC06352	simplex		*GUCA1A*	NM_000409.3	Het	3	c.149C>T	p.(P50L)	Np	Moderately	B	T	Dc	(rs104893968) [90]
CIC07188	simplex		*PROM1*	NM_006017.2	Het	12	c.1354dup	p.(Y452Lfs*13)	Np	-	-	-	-	[71]
			*PROM1*	NM_006017.2	Het	IVS 12	c.1454+2>C	r.(spl?)	Np	Highly	-	-	-	Novel
CIC03241	simplex		*CRX*	NM_000554.4	Het	4	c.564dup	p.(A189Rfs*47)	+	-	-	-	-	Not clear if same mutation as in [69]
CIC07569	simplex		*CRX*	NM_000554.4	Het	IVS 3	c.252+1G>A	r.(spl?)	Np	Highly	-	-	-	Novel

*Ar*: autosomal recessive; *Ad*: autosomal dominant; *Het*: heterozygous; *Ho*: homozygous; *Consang*.: Consanguinity; *Coseg*.: Cosegregation; *Np*: Not possible; *B*: Benign; *T*: Tolerated; *Prd*: Proabably damaging, *Pd*: Possible disease causing; *D*: Damaging; *Dc*: Dicease causing

^a^ personal communication B. Puech

^b^ Sergouniotis P. (2012). *Genotype and phenotypic heterogeneity in autosomal recessive retinal disease*. Ph.D.Thesis. Institute of Ophthalmology, University College London, United Kingdom

^c^ sibling patients. Only one of the 2 siblings (CIC06642) was considered for the mutation prevalence analysis

^d^ these mutations were considered pathogenic with lower confidence because biallelism could not be demonstrated by cosegregation analysis

Among the 21 resolved cases other initially classified sporadic, 5 (23.8 %) harboured heterozygous mutations in genes implicated in ad forms and 16 (76.2 %) harboured homozygous or compound heterozygous mutations in genes implicated in ar cases. We suspected “*de novo”* mutations for all 5 sporadic patients with heterozygous mutations in ad genes. For patient CIC03241, both parents were not clinically affected and did not carry the pathogenic mutation previously reported in Leber Congenital Amaurosis (LCA) (*CRX* het c.564dup p.(A189Rfs*47) (Additional file 2)). Similarly, for CIC03750, both parents were unaffected and did not carry the known pathogenic mutation (*CRX* het c.121C>T, p.(R41W)) (Additional file 2). Unfortunately, we could not perform haplotype analysis around these mutations in the respective families in order to formally exclude non-paternity, in the absence of ethical agreement for such research. For another subject, CIC00597 carrying the known pathogenic variant in *GUCY2D* c.2747T>C, p.(I916T), only the unaffected mother and sister who did not carry the change, were available for genetic analysis (Additional file 2). Patient had lost contact with his father during childhood and no ophthalmic status for him was available to us. For two additional sporadic cases (CIC06352 carrying the known pathogenic variant c.149C>T, p.(P50L) in *GUCA1A* and CIC07569 carrying a novel splice site variant in *CRX* c.252 + 1G>A) no other family members were available to further validate the “*de novo”* character of the mutation.

*Pathogenic mutations in retinal disease genes not classically associated with CCRD.* Table 2 shows pathogenic or likely pathogenic mutations in other IRD genes found in 17 patients (4 sporadic cases, 11 ar and 2 ad cases). A total of 19 known and novel mutations were identified in 12 genes not classically associated with CCRD. These mutations were previously reported in RP and LCA (*C2Orf71* [35, 36]*, MERTK* [37]*, RLBP1* [38]*, EYS* [23, 39]*, NMNAT1* [40–42], *RDH12* [43, 44])*, RP1* [45, previous 4]*, CRB1* [46, 47] and *TULP1* [48])*,* Senior-Loken (*IQCB1)* [49]*,* ad vitreoretinochoroidopathy (*BEST1*) [50] and adRP (*ROM1*) [51]. Although, *TULP1* is not a gene that was frequently associated with CCRD (not classified as such in https://sph.uth.edu/retnet/), a recent article revealed *TULP1* mutation as a novel cause of cone dysfunction [52].

**Table 2:** Summary of 19 patients carrying pathogenic or likely pathogenic mutations in other retinal disease genes

ID	Type	Consang.	Gene	NM	Allele State	Exon	cDNA	Protein	Coseg.	Conservation	Polyphen2	Sift	Mutation Taster	References
*High confidence*														
CIC01571	Ar		*C2Orf71*	NM_001029883.2	Ho	1	c.2950C>T	p.(R984*)	+	-	-	-	-	[36] (RP)
CIC00643	Ar	+	*C2Orf71*	NM_001029883.2	Ho	1	c.1949G>A	p.(W650*)	Np	-	-	-	-	Novel (rs371289954)
CIC03112	Ar	+	*MERTK*	NM_006343.2	Ho	17	c.2214del	p.(C738Wfs*32)	Np	-	-	-	-	[37] (RP)
CIC01242	Ar		*MERTK*	NM_006343.2	Ho	3_19	c.483-?_c.3000+?del	-	+	-	-	-	-	Novel
CIC06514	Ar	+	*RLBP1*	NM_000326.4	Ho	7_9	c.526-?_c.954+?del	-	Np	-	-	-	-	Novel
CIC03953	simplex		*EYS*	NM_001292009.1	Het	11	c.1673G>A	p.(W558*)	+	-	-	-	-	[23] (RP) (rs201823777)
			*EYS*	NM_001292009.1	Het	14	c.2234A>G	p.(N745S)	+	Not	B	-	Poly	[23] (RP) (rs201652272)
CIC05012	simplex		*NMNAT1*	NM_022787.3	Het	5	c.619C>T	p.(R207W)	Np	Weakly	B	D	Dc	[41] (LCA) (rs142968179)
			*NMNAT1*	NM_022787.3	Het	5	c.769G>A	p.(E257K)	Np	Moderately	B	T	Dc	[42] (LCA) (rs150726175)
CIC06499	simplex		*NMNAT1*	NM_022787.3	Het	5	c.619C>T	p.(R207W)	+	Weakly	B	D	Dc	[41] (LCA) (rs142968179)
			*NMNAT1*	NM_022787.3	Het	5	c.769G>A	p.(E257K)	+	Moderately	B	T	Dc	[42] (LCA) (rs150726175)
CIC05394	Ar	+	*RDH12*	NM_152443.2	Ho	8	c.806_810del	p.(A269Gfs*2)	Np	-	-	-	-	[43] (LCA) (rs386834261)
CIC07241	Ar	+	*RDH12*	NM_152443.2	Ho	7	c.464C>T	p.(T155I)	Np	Highly	Pr	D	Dc	[44] (LCA) (rs121434337)
CIC07447	Ar		*RDH12*	NM_152443.2	Het	8	c.806_810del	p.(A269Gfs*2)	+	-	-	-		[43] (LCA) (rs386834261)
			*RDH12*	NM_152443.2	Het	8	c.403A>G	p.(K135E)	+	Highly	Prd	T	Dc	Novel
CIC00953	simplex		*IQCB1*	NM_001023570.2	Het	6	c.424_425del	p.(F142Pfs*5)	+	-	-	-	-	[49] (Senior-Loken/LCA)
			*IQCB1*	NM_001023570.2	Het	8	c.686del	p.(T229Mfs*8)	+	-	-	-	-	Novel
CIC01300	Ar	+	*RP1*	NM_006269.1	Ho	4	c.1719_1723del	p.(S574Cfs*7)	Np	-	-	-	-	[45] (arRP)
CIC05272	Ad		*BEST1*	NM_001139443.1	Het	4	c.76G>A	p.(V26M)	+	Highly	Prd	D	Dc	[50] (ADVIRC) (rs121918289)
CIC01380	Ar	+	*CRB1*	NM_201253.2	Ho	11	c.3994T>G	p.(C1332G)	+	Highly	Prd	D	Dc	Novel (LCA)
CIC00963	Ar	*+*	*TULP1*	NM_003322.4	Ho	11	c.1087G>A	p.(G363R)	+	Highly	Pd	D	Dc	Novel (LCA and arRP)
*Lower confidence*														
CIC05007	Ad		*ROM1*	NM_000327.3	Het	1	c.339del	p.(L114Sfs*8)	+	-	-	-	-	Novel (adRP)
*Most likely not pathogenic*														
CIC05379	simplex		*FSCN2*	NM_001077182.2	Het	1	c.574C>T	p.(R192C)	+	Highly	Prd	D	Dc	Novel (adRP and adMD but questionable) (rs377025075)
CIC05604	simplex		*FSCN2*	NM_001077182.2	Het	1	Partial deletion	-	Np	-	-	-	-	Novel (adRP and adMD but questionable)

*RP*: Retinitis Pigmentosa; *MD*: macular dystrophy; *LCA*: Leber Congenital Amaurosis; *ADVIRC*: Autosomal Dominant Vitreoretinochoroidopathy; *Ad*:autosomal dominant; *Ar*: autosomal recessive; *Consang.*: Consangunity; *Coseg*.: Cosegregation; *Np*: Not possible; *Poly*: Polymorphism ; *Het*: heterozygous; *Ho*: homozygous; *B*: Benign; *T*: Tolerated; *Prd*: Proabably damaging, *Pd*: Possible disease causing; *D*: Damaging; *Dc*: Disease causing

We re-assessed pathogenic prediction for the published and novel variants we identified herein and all of them remain convincing except one on *EYS* (p.N745S) [23, 39], which now appears not conserved with a poor pathogenic prediction profile (Table 2), most likely due to novel sequenced species showing also the S amino acid at position 745.

Mutations in *ROM1* and the implication in retinal disorders are a matter of discussion [51, 53, 54] and larger pedigrees are necessary to validate the pathogenicity of a variant. Therefore, we classified the *ROM1* mutation identified herein with “Lower confidence” (Table 2).

In addition, we identified two patients with variations in *FSCN2*: CIC05379 and CIC05379 carried a heterozygous missense variant c.574C>T, p.(R192C) and a heterozygous partial deletion of exon 1 confirmed by qPCR, respectively (Table 2). For these 2 individuals, we could not confirm segregation of the variant with a disease phenotype in the family (informative family members were not available for genetic testing) and, in view of the low evidence of the role of this gene in the literature, we considered these 2 variants not pathogenic [34, 55, 56].

**Fig. 2:**
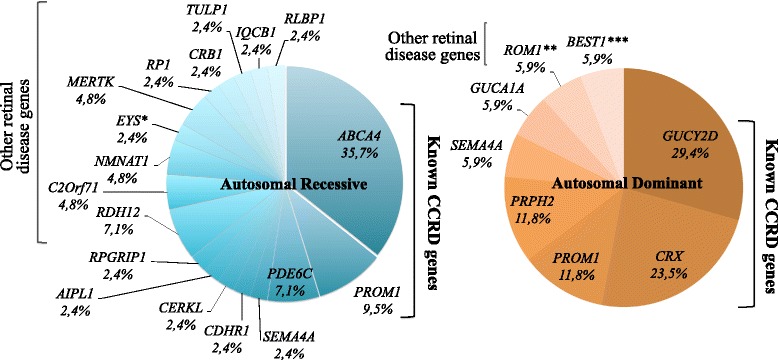
Gene defect spectrum involved in cone and cone-rod dystrophies. Gene defect prevalence in resolved patients with autosomal recessive inheritance (*N* = 42 patients including 20 sporadic cases) and autosomal dominant inheritance (*N* = 17 patients including 5 sporadic cases). * *EYS* disease association with CCRD should be taken with caution. ** the implication of *ROM1* in IRD remains to be elucidated in the future. *** after clinical reassessment, patient’s phenotype was retrospectively more compatible with ADVIRC diagnosis and not CCRD

*Mutation spectrum in patients with ar cases* (Tables 1 and 2, Fig. 2) Taking all gene defects into account, among the 42 patients (27 patients with mutations in known CCRD gene defects and 15 patients with gene defects never associated with CCRD) with 2 or more pathogenic alleles (22 ar cases and 20 sporadic cases), mutations in *ABCA4* were the most prevalent for ar cases (15/42 patients, 35.7 %). For the other 12 cases, mutations were identified in *PROM1* (4/42 patients, 9.5 %), *PDE6C* (3/42 patients, 7.1 %), *SEMA4A* (1/42 patients, 2.4 %), *CDHR1* (1/42 patients, 2.4 %), *CERKL* (1/42 patients, 2.4 %), *AIPL1* (1/42 patients, 2.4 %), *RPGRIP1* (1/42 patients, 2.4 %). For the 15 remaining patients, mutations were identified in genes not classically associated with CCRD (see below). In addition, for 6 patients, only one pathogenic or possibly pathogenic allele was identified implicating *ABCA4* (Additional file 3). Heterozygous status of these variants was confirmed by Sanger sequencing. No second mutation was found by Sanger sequencing poorly covered regions and no evidence of deletion was suspected looking at the coverage data. MLPA did not identify large deletions for these cases. In addition, a single published nonsense variant was identified in *IMPG2* at a heterozygous state in one sporadic case (CIC00680, c.379G>A p.(R127*)) [16] and another novel single variant also heterozygous was found in *CDHR1* (CIC07507, c.1863dupC p.(I622H*fs54)) in an ar case with consanguineous parents (Additional file 3). Sanger sequencing confirmed these two variants. Exonic regions of these 2 genes were well covered with no further evidence of deletion in NGS raw data analysis. Therefore we concluded that these latter 8 cases with heterozygous variants were not yet solved (Additional file 3).

Thirty one index cases with parental consanguinity were included in the study. Among them, 20 were found to carry homozygous mutations, one was compound heterozygous (CIC02712, Table 1), one was carrying a single variant in *ABCA4* (CIC05758, Additional file 3) and another one a single variant in *CDHR1* (CIC07507, Additional file 3) whereas 8 cases did not show any putative pathogenic variant.

*Mutation spectrum in ad cases* (Tables 1 and 2, Fig. 2) Taking all gene defects into account, among the 17 patients with 1 pathogenic or likely pathogenic allele in genes involved in ad retinal dystrophy (12 ad and 5 sporadic cases), *GUCY2D* (5/17 patients, 29.4 %), *CRX* (4/17 patients, 23.5 %), *PROM1* (2/17 patients, 11.8 %) and *PRPH2* (2/17 patients, 11.8 %) were the most prevalent. The others genes *SEMA4A, GUCA1A* were implicated in 5.9 % (1/17 patients) of cases each. For the remaining 2 patients, mutations were identified in genes not classically associated with CCRD (Table 2: *BEST1* c.76G>A, p.(V26M) and *ROM1* c.339del, p.(L114Sfs*8)). Results of co-segregation analysis are shown in Additional file 2.

*Patients with no variant and coverage analysis* For the unsolved probands, we performed coverage analysis and Sanger sequencing of poorly covered regions for genes implicated in CCRD (coverage < 25-fold per base). In doing so, we did not detect any additional pathogenic mutations. However, for 2 patients (CIC06514 and CIC01242 included in Table 2) coverage was found null whatever the depth in regions not classically low-covered by the NGS in the whole cohort. We suspected homozygous deletions of these “not-covered regions”, that were secondary confirmed by the absence of PCR-amplification and the CNVs analysis (see below). CIC06514 showed a homozygous 7.36 kb deletion encompassing the last 3 exons (exons 7 through 9) of *RLBP1* (Table 2). This deletion was previously reported in Retinitis Punctata Albescens (RPA) patients from Morocco [38, 57]. CIC01242 revealed a novel large homozygous deletion of *MERTK* (exons 3 through last exon 19) (Table 2).

Copy Number Variations (CNVs) analysis was also performed to detect large deletion or duplication not detected by NGS technique. By this approach, we confirmed the 2 homozygous deletions previously detected by coverage analysis in *RLBP1* and *MERTK*. We also detected 1 additional novel heterozygous exon 1 deletion of *PRPH2* (CIC03621), which was confirmed by MLPA (Table 1). In addition, while performing MLPA on patients with one single variant on *ABCA4,* we also performed *ABCA4*-MPLA on patients with no known variant and inheritance compatible with ar mode of inheritance. No additional deletion on *ABCA4* was identified applying this technique.

To summarize, in a total of 95 unrelated index patients, targeted NGS identified pathogenic variants in 59 (62.1 %) cases. In addition, in 8 more sporadic cases (8.4 %), only one pathogenic variant in *ABCA4* (6/8) or *IMPG2* (1/8) or *CDHR1* (1/8) was found. Providing that these later cases are not solved, our targeted NGS approach identified in 42 of 78 sporadic and ar (53.8 %) and in 17 of 17 ad (100 %) cases disease causing mutations. Of notes coverage and CNV analysis of NGS raw data was useful to resolve cases with large deletions, for which the first step of NGS analysis did not identify disease causing variants.

### Phenotype and clinical reassessment of patients with mutations in IRD genes not classically associated with CCRD

Medical charts from patients with mutations in genes not classically associated with CCRD were reviewed in order to reassess clinical diagnosis.

**Fig. 3:**
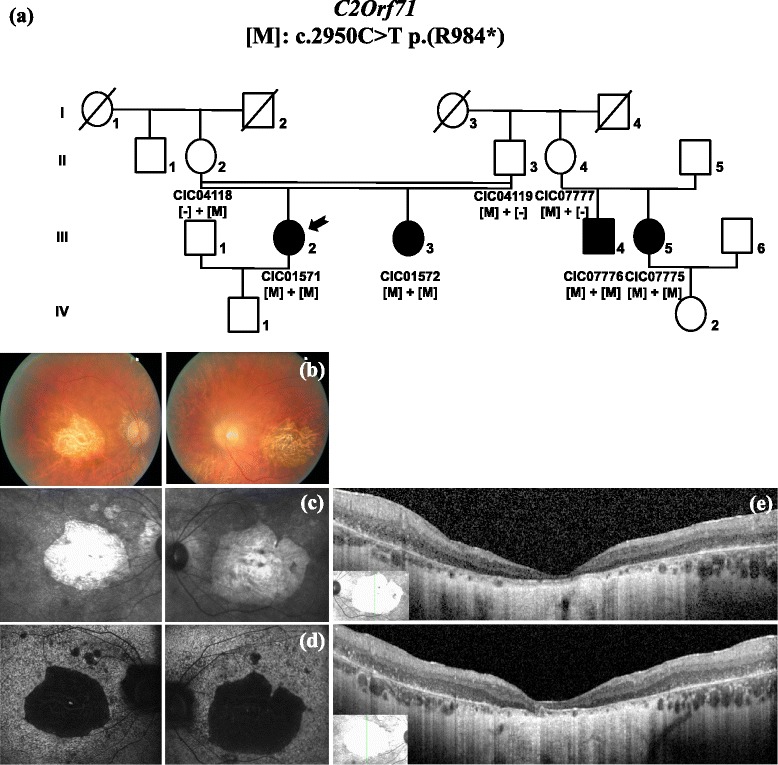
Phenotype of CIC01571 who carries a homozygous nonsense mutation in *C2Orf71,* previously reported in RP. (**a**) Pedigree of family 1932. History of symptoms reveals initial photophobia and bilateral central vision loss. Visual acuity is reduced to hand motion in both eyes. Colour fundus photographs (**b**), infra-red (**c**) and blue autofluorescence imaging (**d**) confirm the predominant central involvement with severe bilateral macular atrophy. SD-OCT (**e**) shows the disappearance of the photoreceptor layers that extends beyond the vascular arcades. ERG responses (not shown) were undetectable from background noise in both scotopic and photopic conditions

CIC01571 and CIC00643 carried homozygous nonsense mutations in *C2Orf71*, among which, one was novel and one was previously reported in RP [36]. For both patients, CRD diagnosis was confirmed according to disease history with primary photophobia and central vision loss associated with predominant central involvement in autofluorescence imaging (Fig. 3). ERG was not useful for differential diagnosis in these cases since responses were undetectable for both scotopic and photopic conditions.

CIC06514 carried a homozygous deletion in *RLBP1* previously described in Moroccan patients with Retinitis Punctata Albescens (RPA) [38, 57]. Our patient was from Algeria, with one sister and one brother affected with RP. He presented high myopia, bilateral keratoconus and surgically removed bilateral congenital cataract. Phenotype was severe with vision reduced to hand motion in both eyes. ERG responses were undetectable for both scotopic and photopic conditions. Fundoscopy showed retinal vessel narrowing, optic disc pallor, peripheral pigment deposits and extensive chorioretinal atrophy. No tiny white dots were observed around the fovea and beyond the vascular arcades as expected in RPA. Phenotype was consistent with both CRD and RP, without any possibility of distinction between the 2 diagnoses at this late stage of the disease. However, according to Dessalces and co-workers, the presence of the white dot-like deposits could be an inconstant finding in RPA, in particular in children or in patients with late-stage disease [57]. Fundus examination of this patient at an earlier stage of the disease would have been useful to formally exclude RPA.

**Fig. 4:**
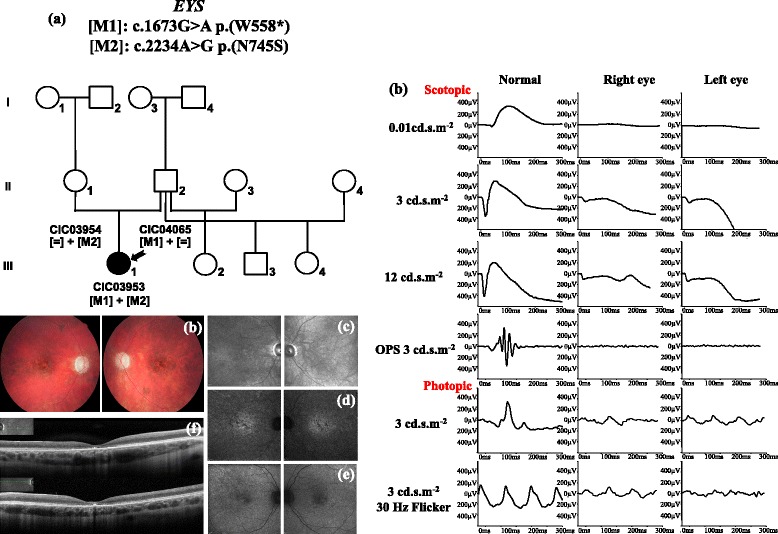
Phenotype of CIC03953 who carries a compound heterozygous mutation in *EYS.* (**a**) Pedigree of family 1819. Visual acuity is 20/50 with −2.50 (−0.25)25° in the right eye and 20/80 with −2.50 (−0.75)170° in the left eye. Kinetic perimetry shows a bilateral central scotoma. Color fundus photographs (**b**) show bilateral optic disc pallor, retinal vessels narrowing and macular pigmentary deposits. Infra-red (**c**), infra-red autofluorescence (**d**) and blue autofluorescence imaging (**e**) highlight macular pigmentary and atrophic changes. SD-OCT (**f**) reveals a predominant central involvement with a disruption of the outer retinal layers limited in the fovea. ERG (**g**) revealed unusual features in association for *EYS* mutations: under scotopic conditions, there was no detectable b-wave in response to a dim (0.01 cd.s.m^−2^) flash while responses to a bright flash showed some reduction of the a-wave but additional b-wave reduction leading to an electronegative waveform. Photopic responses were severely affected in keeping with cone-rod dysfunction with additional inner retinal dysfunction

CIC03953 carried compound heterozygous changes in *EYS* already associated with RP patients [23, 39]. Her fundus showed optic disc pallor, retinal vessel narrowing and pigmentary changes in macular region. ERG revealed unusual features: under scotopic conditions, there was no detectable b-wave in response to a dim (0.01 cd.s.m^−2^) flash while responses to a bright flash showed some reduction of the a-wave but additional b-wave reduction leading to an electronegative waveform. Photopic responses were severely affected in keeping with cone-rod dysfunction with most likely additional inner retinal dysfunction. The SD-OCT revealed a preserved outer nuclear layer thickness but a disruption of the inner segment ellipsoid and interdigitation zones in the foveal region. According to the ERG and SD-OCT findings, atypical CRD diagnosis was retained rather than RP diagnosis (Fig. 4). As above mentioned, although one of the variant carried by this patient is a pathogenic nonsense variant (c.1673G>A p.(W558*)), the second variant is a misssense (c.2234A>G p.(N745S)) with now a questionable pathogenicity. Therefore, although CIC03953 revealed a very peculiar phenotype considering CRD, disease association with *EYS* should be cautious.

CIC05012 and CIC06499 carried compound heterozygous mutations (c.619C>T p.(R207W) + c.769G>A p.(E257K)) in *NMNAT1*, which was recently reported to be responsible for LCA associated with macular coloboma-like atrophic lesions [40–42, 58]. The first patient (CIC05012) was 8 years old and presented sporadic central visual loss since early childhood without nystagmus. ERG showed primarily cone dysfunction consistent with CRD. Fundoscopy revealed bilateral and asymmetrical sharply demarcated macular atrophic lesions, which could be considered as pseudocoloboma. The second patient (CIC06499) had also poor vision since infancy with predominantly cone dysfunction in ERG. Fundus examination showed macular atrophic changes with widespread loss of autofluorescence at the posterior pole but no macular coloboma-like lesion.

CIC00953 was found to be compound heterozygous for two small deletions in *IQCB1* including one already reported in Senior-Loken syndrome [49] and one novel. Mutations in this gene have also been associated with LCA with a need for monitory kidney function due to the variable age of onset of the nephronoptis associated with this gene. Our patient did not have renal insufficiency and displayed more a CCRD phenotype with photophobia, vision reduced to 20/32 in the right eye and 20/100 in the left eye at age 28, central scotoma, cone-rod dysfunction on ERG and moderate autofluorescence changes. Based on this new genetic finding, the patient was advised to regularly monitor her kidney function.

CIC05272 carried a missense mutation in *BEST1* (c.76G>A p.(V26M)) already associated with Autosomal Dominant Vitreoretinochoridopathy (ADVIRC) [50]. Patient’s phenotype was retrospectively more compatible with ADVIRC diagnosis with vitreous fibrillar condensation, circumferential hyperpigmented band in peripheral retina and severely abnormal responses on electrooculogram. Microphtalmos and congenital cataract were observed in other family members, which is consistent with ADVIRC.

CIC01380 carried a novel probably pathogenic homozygous missense mutation in *CRB1*, gene known to be implicated in LCA and RP [47]. Clinical presentation was consistent with CRD. Fundus examination showed bilateral and symmetric bull’s eye maculopathy. Autofluorescence imaging revealed bilateral macular atrophy and ERG showed predominant cone dysfunction. No typical *CRB1* phenotypic features as hypermetropic refractive error, nummular pigmentation at the level of the RPE or increased retinal thickness on OCT were found in this patient [59].

For CIC05007 carrying a mutation in *ROM1*, clinical reassessment confirmed the diagnosis of CRD with predominant cone dysfunction in ERG. Fundus examination revealed optic disc pallor, retinal vessel narrowing and macular pigmentary changes. Autofluorescence pattern was atypical with central hypoautofluorescence surrounded by a large hyperautofluorescent ring with blurred boundaries. As mentioned before the implication of *ROM1* in IRD remains to be elucidated in the future.

The 7 remaining patients carried mutations in *MERTK* (CIC03112, CIC01242), *RP1* (CIC01300), *RDH12* (CIC05394, CIC07241, CIC07447), *TULP1* (CIC00963), which were known to be associated with RP or LCA. For these patients, ERG responses were undetectable in both scotopic and photopic conditions. Distinction between RP and CRD could not be made in the absence retrospective data.

## Discussion

Using NGS approach targeting exons of 123 genes known to be implicated IRDs in a total of 95 unrelated index patients, we could identify likely pathogenic variants in 59 (62.1 %) cases, including those with high and lower confidence. Being more stringent excluding the cases for whom no family members were available to demonstrate biallelism, we would obtain a detection rate of 52 % (49/95). In addition, in 8 more sporadic cases (8.4 %) only one pathogenic variant was found including 6 in *ABCA4*. Of notes coverage and CNV analysis of NGS raw data was useful to resolve cases with large deletions, for which the first step of NGS analysis did not identify disease causing variants. Providing that ar cases with only one variant are not solved, our targeted NGS approach identified in 42 of 78 sporadic and ar (53.8 %) and in 17 of 17 ad (100 %) cases disease causing mutations. This mutation detection rate difference between ar and ad cases may be explained by the fact that our panel targets all genes involved in adCCRD identified so far but is less comprehensive for arCCRD-associated genes (i.e. at least 3 novel genes were not included *RAB28, C21Orf2* and *TTLL5)*. It also reinforces the genetic heterogeneity of arCCRD and may also suggest additional gene defects to be discovered in this genetic group which may not be the case for adCCRD.

To our knowledge, this is the largest NGS panel of known IRD genes applied to CCRD patients. In a recent study, Huang et al used a similar approach including 25 associated-CRD genes to investigate 47 CRD Chinese families [21]. All patients had previously been excluded for mutations in major known genes (*CRX, GUCY2D, GUCA1A, PRPH2, KCNV2*) and for reported mutations in 17 other CRD genes [60]. Their mutation detection rate was 21 % (10/47 families), with 14 potential pathogenic mutations identified, of which 9 were novel. The current study provides a more comprehensive tool, since it screens 123 genes implicated in various IRDs with different inheritance patterns. The detection mutation rate is relatively high (62.1 %) and similar to those obtained for RP [19]. Applying NGS to our retinal disease panel, 33 known and 35 novel mutations were detected in different patients.

Identification of these mutations reaffirms the clinical and genetic heterogeneity of CCRD, and further expands the mutation spectrum of CCRD. Overall in this cohort, the prevalence of known genes was 15.8 % for *ABCA4* (15/95 patients), 6.3 % for *PROM1* (6/95 patients), 5.3 % for *GUCY2D* (5/95 patients), 4.2 % for *CRX* (4/95 patients), 3.2 % for *PDE6C* (3/95 patients), 2.1 % each for *SEMA4A* and *PRPH2* (2/95 patients each), 1 % each for *CDHR1, CERKL, GUCA1A, RPGRIP1, AIPL1* (1/95 patients each). These results confirm that mutations in *ABCA4* are the most prevalent gene defect implicated in CCRD. Not counting patients with suspected or confirmed ad transmission (17 patients), pathogenic or likely pathogenic *ABCA4* mutations were found in 15/78 ar and sporadic CCRD patients (19.2 %). In addition, 6 patients carried at least 1 *ABCA4* mutation (7.7 %). This rate is similar to those described in the literature. Using direct sequencing techniques, Maugeri et al found mutations in both *ABCA4* alleles in 6/20 ar/simplex cases (30 %) [9]. In a study including 54 ar or simplex CRD patients, Klevering et al found homozygous or compound heterozygous mutations in *ABCA4* in 9 patients (16 %) [61]. Kitiratschky and colleagues identified *ABCA4* mutations on both alleles in 25 % of cases (16/64 patients with arCCRD) [62]. Most of these previous studies included smaller cohorts so that *ABCA4* prevalence could have been over estimated. Similarly to previous studies, a significant proportion of patients carried only one pathogenic allele. MPLA did not reveal any large deletions or genomic rearrangement mutations in these patients in keeping with the literature [63, 64]. Braun and co-workers were first to provide evidence for the possibility of intronic variants near rare alternate splice junctions that could be pathogenic by increasing the probability of mis-splicing at these sites [65]. Deep intronic mutations leading to truncated proteins have also been described in Usher syndrome and LCA [66, 67]. This need to be further investigated in our 6 patients. Another hypothesis is that single heterozygous variants found in *ABCA4* are not causative in these cases. The same reasoning holds true for the patients carrying a single pathogenic allele in *IMPG2* or *CDHR1.* Indeed, the presence of additional pathologic mutations in genes different from the causative gene in IRD patients is a frequent event that has been highlighted by high throughput sequencing. Implication of these variants in pathogenicity is unknown. Some genes could play a modifying role on the expression or not of the disease. Only functional studies could help to better understand these potential interactions.

As previously reported, mutations in *GUCY2D* are the major gene defect implicated in ad CCRD, which was found in 5/17 ad CCRD patients (29.4 %) in this study [11]. Sanger sequencing of German families with 10 ad CCRD found similar results with *GUCY2D* mutations in 25 % of cases [68]. In the same study, prevalence of *CRX* and *PROM1* were low (4 % and 2 % respectively) compared with our results (2/17 for *PROM1*, 4/17 for *CRX*, 11.7 % and 23.5 %, respectively).

Furthermore, inheritance pattern could be better defined for 25 sporadic cases (2 of the 27 initial sporadic cases revealed ad transmission after clinical investigation of the family members), who were predominantly ar (20/25 patients, 80 %). The remaining 5 patients showed mutations in ad genes (CIC03241, CIC03750 and CIC07569 in *CRX,* CIC00597 in *GUCY2D,* CIC06352 in *GUCA1A* of which 2 were novel). Regions carrying the 3 *CRX* mutations were repetitive and were therefore likely to be the site of neomutations. The “*de novo*” nature of all these mutations could only be confirmed for 2 patients whose parents did not carry the pathogenic mutation (c.564dup p.(A189Rfs*47) and c.121C>T p.(R41W) in *CRX* previously reported in LCA [69, 70]. We actually could not perform haplotype analysis around these mutations for these families in order to formally exclude non-paternity, in the absence of ethical agreement for this research. In the 3 other cases, we did not manage to genetically investigate their parents (DNA was only available in 1 out of 2 parents for 1 patient and not available for the others) and clinically, in order to conclude. These results should be taken into consideration for patient management since it could modify genetic counselling. Further studies are needed to better estimate the prevalence of pathogenic neomutations in inherited retinal disorders.

This approach can revisit mode of inheritance of gene defects in CCRD and identify new phenotype-genotype associations. As previously reported, mutations in *PROM1* are known to be associated with both recessive and dominant inheritance. According to Pras et al, missense mutations (c.1117C>T p.(R373C) in our cohort) result in a mutant protein that exerts a dominant negative effect explaining the ad inheritance. In contrast, severe frameshift mutations (c.1354dup p.(Y452Lfs*13); c.7dup p.(L3P*28)) most likely abolish the function of one allele with the other functioning allele being sufficient to maintain normal retinal activity. Symptoms develop only when both alleles are not expressed [71]. Similarly, mutations in *AIPL1* are already known to be implicated in both ar LCA and ad CRD [72]. A recent study identified two homozygous mutations in *AIPL1* (c.773G>C (p.R258P) and c.465G>T (p.(H93_Q155del)) in two Pakistani families, which co-segregated with disease phenotype that could correspond to ar LCA or ar RP [73]. In 1 patient of our cohort, 2 novel likely pathogenic mutations were identified in exon 5 of *AIPL1* (het c.769C>T p.(L257F) + het c.767T>G p.(I256S)), one carried by the unaffected father and one by the unaffected mother. This would suggest that *AIPL1* mutations can also lead to ar CRD although no additional affected member was available to confirm pathogenicity. Lastly, one patient with severe simplex CRD carried a novel homozygous nonsense mutation in *SEMA4A* (c.241C>T p.(R81*)), known to be responsible for ad CRD or ad RP. Although no other family members were available for co-segregation analysis, this truncating mutation is likely causative and would also expand the spectrum of *SEMA4A* to CRD with ar inheritance. *SEMA4A* mutations previously described in ad RP or ad CRD were missense mutations localized between codon number 345 and 713 [74]. In our case, the mutation was homozygous, nonsense and localized earlier on the protein (codon number 81). We hypothesized that in ad cases, mutations of one allele lead to abnormal product that exerts a dominant negative effect on the normal product from the non mutated allele. On the opposite, in ar cases, mutations are more severe (nonsense, earlier on the genomic sequence) with product degradation due to nonsense-mediated RNA decay, leading to haploinsufficiency. In the last cases, mutations in both alleles are necessary to develop the disease. However, this hypothesis needs to be confirmed by further functional analysis. Another hypothesis is that the mutation p.(R81*) at the heterozygous state is enough to be pathogenic. However, one must be cautious before concluding on this first ar mutation in *SEMA4A*, since cosegregation analysis was not possible to document the biallelism in association with the disease.

This approach also allowed us to establish new genotype-phenotype correlations or to refine clinical diagnosis. In this cohort, 17 patients showed inconsistency between molecular diagnosis and initial clinical diagnosis. Clinical reassessment was performed to resolve these cases. For one patient, clinical diagnosis was modified based on additional clinical data (CIC05272: ADVIRC). For another patient, (CIC00953: *IQCB1*) genetic analysis led to monitoring of renal function with a significant consequence on patient management. For 7/17 patients, CRD diagnosis was confirmed, suggesting new genotype-phenotype correlations for the following genes.

### *C2Orf71*

CRD diagnosis was confirmed for both CIC00643 and CIC01571, suggesting that *C2Orf71* is not only responsible for RP (≈1 % of cases according to Audo et al [36]) but also for CRD, with an estimated prevalence of 2.5 % in this cohort (2/78 ar or sporadic patients). This is in accordance with findings by Collin et al who previously reported two patients with ERG recordings suggesting more affected cone than rod function, although both photoreceptor functions were severely damaged [35].

### *EYS*

Diagnosis of atypical CRD was retained for CIC03953. A recent report described the case of a Japanese patient with ar CRD and compound heterozygous truncating mutations in *EYS* [75]. With our additional case, we confirmed that the phenotypic spectrum of *EYS* mutations can be extended to ar CRD, but we also described a new *EYS*-related phenotype, characterized by a scotopic electronegative waveform in ERG. However, as mentioned before, the pathogenic character of the missense mutation in this patient needs to be confirmed.

### *NMNAT1*

Although clinical presentations of 2 patients (particularly CIC05012) were not highly suggestive of *NMNAT1*-mutant phenotype, Chiang and colleagues have already reported in their cohort 1 patient with confirmed CRD diagnosis who carried a compound heterozygous mutation in *NMNAT1*, including the most frequent variant c.769G>A p.(E257K) [42]. According to these observations, the clinical spectrum of *NMNAT1* could be expanded from LCA to CRD. However, these findings are not consistent with a recent report that did not identify disease-causing *NMNAT1* mutations among 108 CRD patients [76]. Furthermore, the interpretation of the most frequent variant (c.769G>A p.(E257K)) should be interpreted with caution since it was recently found homozygous in a subject with no ocular abnormalities, suggesting that this variant may not be fully penetrant [77]. Siemiatkowska et al hypothesized that this hypomorphic variant causes LCA in combination with a more severe *NMNAT1* variant. Incomplete penetrance may be due to *trans*- or *cis*-acting elements, which could influence its expression. Another hypothesis is that this variant is not pathogenic but rather is in linkage disequilibrium with an undetected mutation.

### *CRB1*

Although CIC01380 did not show common phenotypic features associated with *CRB1*, Henderson et al reported a high clinical variability in IRDs due to *CRB1* mutations [59]. Furthermore, as in our patient, they observed macular atrophy in 29 % of cases in a cohort of 34 *CRB1*-associated RP and LCA. In the same cohort, 3 patients showed more a CRD phenotype than RP with early onset decreased vision, generalized photoreceptor dysfunction and evidence of more severe macular dysfunction. No macular atrophy was reported in these 3 cases. According to these findings, CRD should be considered part of the clinical spectrum associated with *CRB1* mutation.

### *TULP1*

We did not consider this gene as known to be associated with CCRD since it was not listed in Retnet database. However, using a homozygosity mapping approach in a cohort of 159 CCRD, Roosing et al. [52] recently reported the cases of 2 unrelated patients with predominantly cone-mediated phenotype (1 with CD and 1 with CRD) carrying a novel homozygous missense mutation (p.R420S) in *TULP1,* predicted as pathogenic. Both patients were sporadic cases from consanguineous parents with heterozygous status for the mutation so no cosegregation analysis with the disease could be performed [52]. Our additional case confirms that the disease spectrum of *TULP1* mutations extends from early-onset RP to cone-dominated disease. Further research is required to elucidate the molecular mechanisms associated with the different diseases associated with *TULP1* mutations and will aid a better understanding of the function of *TULP1* in cones and rods.

### *ROM1*

CIC05007 and his affected sibling carried a novel heterozygous frameshift deletion in *ROM1* (c.339del p.(L114Sfs*8)). This gene was initially reported in digenic RP with *PRPH2* mutation [78] and then implicated in a family with monogenic RP (c.339dupG p.(L114Afs*18)) [79]. However pathogenicity of mutations in *ROM1* is still a matter of debate. In a recent study, Jinda and colleagues found the known heterozygous *ROM1* variant p.(L114Afs*18) in 2/240 normal controls, and in 1 ar RP with likely causative mutation already detected in *C8Orf37*. In our patient, the selected *ROM1* mutation is considered to be causative with low confidence. Further co-segregation analysis could document the association between CRD and the genetic defect.

By using targeted NGS, 36 patients (37.9 %) were still genetically unresolved and could be further investigated with whole exome sequencing (WES). In our experience, coverage analysis of genes of interest by direct sequencing of low-covered regions should be systematically performed, particularly in regions with null coverage whatever the depth. This step is essential before WES, in order to exclude rare mutations in low-covered regions and more importantly, homozygous exonic deletions as found in this study.

## Conclusions

In summary, in this study, we applied NGS to a cohort of 96 CCRD patients using a comprehensive panel of 123 IRD genes. Using this approach, prevalence of gene defects underlying ad CCRD and ar CCRD were determined for the first time in a large cohort. Novel mutations were found, expanding the mutations spectrum of CCRD. New genotype-phenotype correlations were identified, confirming that genetic of IRD is complex with an extensive overlap between CCRD and other retinal diseases.

## Additional files

Additional file 1:
**Panel of 123 known genes responsible for retinal disease with photoreceptor degeneration.**
Additional file 2:
**Cosegregation analysis of pathogenic or likely pathogenic mutations.**
Additional file 3:
**Summary of 8 patients carrying heterozygous pathogenic or possibly pathogenic mutations in** ***ABCA4,*** ***IMPG2*** **and** ***CDHR1.***

## References

[1] Hamel CP (2007). Cone rod dystrophies. Orphanet J Rare Dis.

[2] Thiadens AAHJ, Phan TML, Zekveld-Vroon RC, Leroy BP, van den Born LI, Hoyng CB (2012). Clinical course, genetic etiology, and visual outcome in cone and cone-rod dystrophy. Ophthalmology.

[3] Michaelides M, Hardcastle AJ, Hunt DM, Moore AT (2006). Progressive cone and cone-rod dystrophies: phenotypes and underlying molecular genetic basis. Surv Ophthalmol.

[4] Audo I, Mohand-Saïd S, Dhaenens C-M, Germain A, Orhan E, Antonio A (2012). RP1 and autosomal dominant rod-cone dystrophy: novel mutations, a review of published variants, and genotype-phenotype correlation. Hum Mutat.

[5] Jalili IK, Smith NJ (1988). A progressive cone-rod dystrophy and amelogenesis imperfecta: a new syndrome. J Med Genet.

[6] Aleman TS, Cideciyan AV, Volpe NJ, Stevanin G, Brice A, Jacobson SG (2002). Spinocerebellar ataxia type 7 (SCA7) shows a cone-rod dystrophy phenotype. Exp Eye Res.

[7] Littink KW, Koenekoop RK, van den Born LI, Collin RWJ, Moruz L, Veltman JA (2010). Homozygosity mapping in patients with cone-rod dystrophy: novel mutations and clinical characterizations. Invest Ophthalmol Vis Sci.

[8] Roosing S, Thiadens AAHJ, Hoyng CB, Klaver CCW, Den Hollander AI, Cremers FPM (2014). Causes and consequences of inherited cone disorders. Prog Retin Eye Res.

[9] Maugeri A, Klevering BJ, Rohrschneider K, Blankenagel A, Brunner HG, Deutman AF (2000). Mutations in the ABCA4 (ABCR) gene are the major cause of autosomal recessive cone-rod dystrophy. Am J Hum Genet.

[10] Kelsell RE, Gregory-Evans K, Payne AM, Perrault I, Kaplan J, Yang RB (1998). Mutations in the retinal guanylate cyclase (RETGC-1) gene in dominant cone-rod dystrophy. Hum Mol Genet.

[11] Kitiratschky VBD, Wilke R, Renner AB, Kellner U, Vadalà M, Birch DG (2008). Mutation analysis identifies GUCY2D as the major gene responsible for autosomal dominant progressive cone degeneration. Invest Ophthalmol Vis Sci.

[12] Demirci FYK, Rigatti BW, Wen G, Radak AL, Mah TS, Baic CL (2002). X-linked cone-rod dystrophy (locus COD1): identification of mutations in RPGR exon ORF15. Am J Hum Genet.

[13] Yang Z, Peachey NS, Moshfeghi DM, Thirumalaichary S, Chorich L, Shugart YY (2002). Mutations in the RPGR gene cause X-linked cone dystrophy. Hum Mol Genet.

[14] Audo I, Bujakowska KM, Léveillard T, Mohand-Saïd S, Lancelot M-E, Germain A (2012). Development and application of a next-generation-sequencing (NGS) approach to detect known and novel gene defects underlying retinal diseases. Orphanet J Rare Dis.

[15] Simpson DA, Clark GR, Alexander S, Silvestri G, Willoughby CE (2011). Molecular diagnosis for heterogeneous genetic diseases with targeted high-throughput DNA sequencing applied to retinitis pigmentosa. J Med Genet.

[16] Neveling K, Collin RWJ, Gilissen C, van Huet RAC, Visser L, Kwint MP (2012). Next-generation genetic testing for retinitis pigmentosa. Hum Mutat.

[17] O’Sullivan J, Mullaney BG, Bhaskar SS, Dickerson JE, Hall G, O’Grady A (2012). A paradigm shift in the delivery of services for diagnosis of inherited retinal disease. J Med Genet.

[18] Shanks ME, Downes SM, Copley RR, Lise S, Broxholme J, Hudspith KA (2013). Next-generation sequencing (NGS) as a diagnostic tool for retinal degeneration reveals a much higher detection rate in early-onset disease. Eur J Hum Genet EJHG.

[19] Glöckle N, Kohl S, Mohr J, Scheurenbrand T, Sprecher A, Weisschuh N (2014). Panel-based next generation sequencing as a reliable and efficient technique to detect mutations in unselected patients with retinal dystrophies. Eur J Hum Genet EJHG.

[20] Wang F, Wang H, Tuan H-F, Nguyen DH, Sun V, Keser V (2014). Next generation sequencing-based molecular diagnosis of retinitis pigmentosa: identification of a novel genotype-phenotype correlation and clinical refinements. Hum Genet.

[21] Huang L, Zhang Q, Li S, Guan L, Xiao X, Zhang J (2013). Exome sequencing of 47 chinese families with cone-rod dystrophy: mutations in 25 known causative genes. PLoS One.

[22] Souied E, Segues B, Ghazi I, Rozet JM, Chatelin S, Gerber S (1997). Severe manifestations in carrier females in X linked retinitis pigmentosa. J Med Genet.

[23] Audo I, Sahel J-A, Mohand-Saïd S, Lancelot M-E, Antonio A, Moskova-Doumanova V (2010). EYS is a major gene for rod-cone dystrophies in France. Hum Mutat.

[24] Aboshiha J, Dubis AM, Carroll J, Hardcastle AJ, Michaelides M. The cone dysfunction syndromes. Br J Ophthalmol. 2015. doi: 10.1136/bjophthalmol-2014-306505 [Epub ahead of print]. 10.1136/bjophthalmol-2014-306505 PMC4717370 25770143

[25] Vincent A, Robson AG, Holder GE (2013). Pathognomonic (diagnostic) ERGs. A review and update. Retina Phila Pa.

[26] Audo I, Robson AG, Holder GE, Moore AT (2008). The negative ERG: clinical phenotypes and disease mechanisms of inner retinal dysfunction. Surv Ophthalmol.

[27] Parry DA, Mighell AJ, El-Sayed W, Shore RC, Jalili IK, Dollfus H (2009). Mutations in CNNM4 cause Jalili syndrome, consisting of autosomal-recessive cone-rod dystrophy and amelogenesis imperfecta. Am J Hum Genet.

[28] Abu-Safieh L, Alrashed M, Anazi S, Alkuraya H, Khan AO, Al-Owain M (2013). Autozygome-guided exome sequencing in retinal dystrophy patients reveals pathogenetic mutations and novel candidate disease genes. Genome Res.

[29] Roosing S, Rohrschneider K, Beryozkin A, Sharon D, Weisschuh N, Staller J (2013). Mutations in RAB28, encoding a farnesylated small GTPase, are associated with autosomal-recessive cone-rod dystrophy. Am J Hum Genet.

[30] Sergouniotis PI, Chakarova C, Murphy C, Becker M, Lenassi E, Arno G (2014). Biallelic variants in TTLL5, encoding a tubulin glutamylase, cause retinal dystrophy. Am J Hum Genet.

[31] Adzhubei IA, Schmidt S, Peshkin L, Ramensky VE, Gerasimova A, Bork P (2010). A method and server for predicting damaging missense mutations. Nat Methods.

[32] Kumar P, Henikoff S, Ng PC (2009). Predicting the effects of coding non-synonymous variants on protein function using the SIFT algorithm. Nat Protoc.

[33] Schwarz JM, Rödelsperger C, Schuelke M, Seelow D (2010). MutationTaster evaluates disease-causing potential of sequence alterations. Nat Methods.

[34] Jin Z-B, Mandai M, Homma K, Ishigami C, Hirami Y, Nao-I N (2008). Allelic copy number variation in FSCN2 detected using allele-specific genotyping and multiplex real-time PCRs. Invest Ophthalmol Vis Sci.

[35] Collin RWJ, Safieh C, Littink KW, Shalev SA, Garzozi HJ, Rizel L (2010). Mutations in C2ORF71 cause autosomal-recessive retinitis pigmentosa. Am J Hum Genet.

[36] Audo I, Lancelot M-E, Mohand-Saïd S, Antonio A, Germain A, Sahel J-A (2011). Novel C2orf71 mutations account for ∼ 1 % of cases in a large French arRP cohort. Hum Mutat.

[37] Tschernutter M, Jenkins SA, Waseem NH, Saihan Z, Holder GE, Bird AC (2006). Clinical characterisation of a family with retinal dystrophy caused by mutation in the Mertk gene. Br J Ophthalmol.

[38] Humbert G, Delettre C, Sénéchal A, Bazalgette C, Barakat A, Bazalgette C (2006). Homozygous deletion related to Alu repeats in RLBP1 causes retinitis punctata albescens. Invest Ophthalmol Vis Sci.

[39] Barragán I, Borrego S, Pieras JI, González-del Pozo M, Santoyo J, Ayuso C (2010). Mutation spectrum of EYS in Spanish patients with autosomal recessive retinitis pigmentosa. Hum Mutat.

[40] Koenekoop RK, Wang H, Majewski J, Wang X, Lopez I, Ren H (2012). Mutations in NMNAT1 cause Leber congenital amaurosis and identify a new disease pathway for retinal degeneration. Nat Genet.

[41] Perrault I, Hanein S, Zanlonghi X, Serre V, Nicouleau M, Defoort-Delhemmes S (2012). Mutations in NMNAT1 cause Leber congenital amaurosis with early-onset severe macular and optic atrophy. Nat Genet.

[42] Chiang P-W, Wang J, Chen Y, Fu Q, Zhong J, Chen Y (2012). Exome sequencing identifies NMNAT1 mutations as a cause of Leber congenital amaurosis. Nat Genet.

[43] Janecke AR, Thompson DA, Utermann G, Becker C, Hübner CA, Schmid E (2004). Mutations in RDH12 encoding a photoreceptor cell retinol dehydrogenase cause childhood-onset severe retinal dystrophy. Nat Genet.

[44] Thompson DA, Janecke AR, Lange J, Feathers KL, Hübner CA, McHenry CL (2005). Retinal degeneration associated with RDH12 mutations results from decreased 11-cis retinal synthesis due to disruption of the visual cycle. Hum Mol Genet.

[45] El Shamieh S, Boulanger-Scemama E, Lancelot M-E, Antonio A, Démontant V, Condroyer C (2015). Targeted Next Generation Sequencing Identifies Novel Mutations in RP1 as a Relatively Common Cause of Autosomal Recessive Rod-Cone Dystrophy. Bio Med Res Int.

[46] Lotery AJ, Jacobson SG, Fishman GA, Weleber RG, Fulton AB, Namperumalsamy P (2001). Mutations in the CRB1 gene cause Leber congenital amaurosis. Arch Ophthalmol.

[47] Bujakowska K, Audo I, Mohand-Saïd S, Lancelot M-E, Antonio A, Germain A (2012). CRB1 mutations in inherited retinal dystrophies. Hum Mutat.

[48] Gu S, Lennon A, Li Y, Lorenz B, Fossarello M, North M (1998). Tubby-like protein-1 mutations in autosomal recessive retinitis pigmentosa. Lancet.

[49] Otto EA, Loeys B, Khanna H, Hellemans J, Sudbrak R, Fan S (2005). Nephrocystin-5, a ciliary IQ domain protein, is mutated in Senior-Loken syndrome and interacts with RPGR and calmodulin. Nat Genet.

[50] Yardley J, Leroy BP, Hart-Holden N, Lafaut BA, Loeys B, Messiaen LM (2004). Mutations of VMD2 splicing regulators cause nanophthalmos and autosomal dominant vitreoretinochoroidopathy (ADVIRC). Invest Ophthalmol Vis Sci.

[51] Bascom RA, Liu L, Heckenlively JR, Stone EM, McInnes RR (1995). Mutation analysis of the ROM1 gene in retinitis pigmentosa. Hum Mol Genet.

[52] Roosing S, van den Born LI, Hoyng CB, Thiadens AAHJ, de Baere E, Collin RWJ (2013). Maternal uniparental isodisomy of chromosome 6 reveals a TULP1 mutation as a novel cause of cone dysfunction. Ophthalmology.

[53] Dryja TP, Hahn LB, Kajiwara K, Berson EL (1997). Dominant and digenic mutations in the peripherin/RDS and ROM1 genes in retinitis pigmentosa. Invest Ophthalmol Vis Sci.

[54] Martínez-Mir A, Vilela C, Bayés M, Valverde D, Dain L, Beneyto M (1997). Putative association of a mutant ROM1 allele with retinitis pigmentosa. Hum Genet.

[55] Gamundi MJ, Hernan I, Maseras M, Baiget M, Ayuso C, Borrego S (2005). Sequence variations in the retinal fascin FSCN2 gene in a Spanish population with autosomal dominant retinitis pigmentosa or macular degeneration. Mol Vis.

[56] Zhang Q, Li S, Xiao X, Jia X, Guo X (2007). The 208delG mutation in FSCN2 does not associate with retinal degeneration in Chinese individuals. Invest Ophthalmol Vis Sci.

[57] Dessalces E, Bocquet B, Bourien J, Zanlonghi X, Verdet R, Meunier I (2013). Early-onset foveal involvement in retinitis punctata albescens with mutations in RLBP1. JAMA Ophthalmol.

[58] Falk MJ, Zhang Q, Nakamaru-Ogiso E, Kannabiran C, Fonseca-Kelly Z, Chakarova C (2012). NMNAT1 mutations cause Leber congenital amaurosis. Nat Genet.

[59] Henderson RH, Mackay DS, Li Z, Moradi P, Sergouniotis P, Russell-Eggitt I (2011). Phenotypic variability in patients with retinal dystrophies due to mutations in CRB1. Br J Ophthalmol.

[60] Huang L, Li S, Xiao X, Jia X, Wang P, Guo X (2013). Screening for variants in 20 genes in 130 unrelated patients with cone-rod dystrophy. Mol Med Rep.

[61] Klevering BJ, Yzer S, Rohrschneider K, Zonneveld M, Allikmets R, van den Born LI (2004). Microarray-based mutation analysis of the ABCA4 (ABCR) gene in autosomal recessive cone-rod dystrophy and retinitis pigmentosa. Eur J Hum Genet EJHG.

[62] Kitiratschky VBD, Grau T, Bernd A, Zrenner E, Jägle H, Renner AB (2008). ABCA4 gene analysis in patients with autosomal recessive cone and cone rod dystrophies. Eur J Hum Genet EJHG.

[63] Aguirre-Lamban J, Riveiro-Alvarez R, Maia-Lopes S, Cantalapiedra D, Vallespin E, Avila-Fernandez A (2009). Molecular analysis of the ABCA4 gene for reliable detection of allelic variations in Spanish patients: identification of 21 novel variants. Br J Ophthalmol.

[64] Yatsenko AN, Shroyer NF, Lewis RA, Lupski JR (2003). An ABCA4 genomic deletion in patients with Stargardt disease. Hum Mutat.

[65] Braun TA, Mullins RF, Wagner AH, Andorf JL, Johnston RM, Bakall BB (2013). Non-exomic and synonymous variants in ABCA4 are an important cause of Stargardt disease. Hum Mol Genet.

[66] Steele-Stallard HB, Le Quesne SP, Lenassi E, Luxon LM, Claustres M, Roux A-F (2013). Screening for duplications, deletions and a common intronic mutation detects 35 % of second mutations in patients with USH2A monoallelic mutations on Sanger sequencing. Orphanet J Rare Dis.

[67] Mackay DS, Borman AD, Sui R, van den Born LI, Berson EL, Ocaka LA (2013). Screening of a large cohort of leber congenital amaurosis and retinitis pigmentosa patients identifies novel LCA5 mutations and new genotype-phenotype correlations. Hum Mutat.

[68] Kohl S, Kitiratschky V, Papke M, Schaich S, Sauer A, Wissinger B (2012). Genes and mutations in autosomal dominant cone and cone-rod dystrophy. Adv Exp Med Biol.

[69] Stone EM (2007). Leber congenital amaurosis - a model for efficient genetic testing of heterogeneous disorders: LXIV Edward Jackson Memorial Lecture. Am J Ophthalmol.

[70] Swain PK, Chen S, Wang QL, Affatigato LM, Coats CL, Brady KD (1997). Mutations in the cone-rod homeobox gene are associated with the cone-rod dystrophy photoreceptor degeneration. Neuron.

[71] Pras E, Abu A, Rotenstreich Y, Avni I, Reish O, Morad Y (2009). Cone-rod dystrophy and a frameshift mutation in the PROM1 gene. Mol Vis.

[72] Sohocki MM, Perrault I, Leroy BP, Payne AM, Dharmaraj S, Bhattacharya SS (2000). Prevalence of AIPL1 mutations in inherited retinal degenerative disease. Mol Genet Metab.

[73] Jin C, Jiao X, Li L, Bushra T, Naeem MA (2014). AIPL1 implicated in the pathogenesis of two cases of autosomal recessive retinal degeneration. Mol Vis.

[74] Abid A, Ismail M, Mehdi SQ, Khaliq S (2006). Identification of novel mutations in the SEMA4A gene associated with retinal degenerative diseases. J Med Genet.

[75] Katagiri S, Akahori M, Hayashi T, Yoshitake K, Gekka T, Ikeo K (2014). Autosomal recessive cone-rod dystrophy associated with compound heterozygous mutations in the EYS gene. Doc Ophthalmol Adv Ophthalmol.

[76] Siemiatkowska AM, van den Born LI, van Genderen MM, Bertelsen M, Zobor D, Rohrschneider K (2014). Novel compound heterozygous NMNAT1 variants associated with Leber congenital amaurosis. Mol Vis.

[77] Siemiatkowska AM, Schuurs-Hoeijmakers JHM, Bosch DGM, Boonstra FN, Riemslag FCC, Ruiter M (2014). Nonpenetrance of the Most Frequent Autosomal Recessive Leber Congenital Amaurosis Mutation in NMNAT1. JAMA Ophthalmol.

[78] Kajiwara K, Berson EL, Dryja TP (1994). Digenic retinitis pigmentosa due to mutations at the unlinked peripherin/RDS and ROM1 loci. Science.

[79] Sakuma H, Inana G, Murakami A, Yajima T, Weleber RG, Murphey WH (1995). A heterozygous putative null mutation in ROM1 without a mutation in peripherin/RDS in a family with retinitis pigmentosa. Genomics.

[80] Jin X, Qu LH, Meng XH, Xu HW, Yin ZQ (2014). Detecting genetic variations in hereditary retinal dystrophies with next-generation sequencing technology. Mol Vis.

[81] Allikmets R, Shroyer NF, Singh N, Seddon JM, Lewis RA, Bernstein PS (1997). Mutation of the Stargardt disease gene (ABCR) in age-related macular degeneration. Science.

[82] Simonelli F, Testa F, de Crecchio G, Rinaldi E, Hutchinson A, Atkinson A (2000). New ABCR mutations and clinical phenotype in Italian patients with Stargardt disease. Invest Ophthalmol Vis Sci.

[83] Stenirri S, Alaimo G, Manitto MP, Brancato R, Ferrari M, Cremonesi L (2008). Are microarrays useful in the screening of ABCA4 mutations in Italian patients affected by macular degenerations?. Clin Chem Lab Med CCLM FESCC.

[84] Huang WC, Cideciyan AV, Roman AJ, Sumaroka A, Sheplock R, Schwartz SB (2014). Inner and outer retinal changes in retinal degenerations associated with ABCA4 mutations. Invest Ophthalmol Vis Sci.

[85] Rozet JM, Gerber S, Souied E, Perrault I, Châtelin S, Ghazi I (1998). Spectrum of ABCR gene mutations in autosomal recessive macular dystrophies. Eur J Hum Genet EJHG.

[86] Wells J, Wroblewski J, Keen J, Inglehearn C, Jubb C, Eckstein A (1993). Mutations in the human retinal degeneration slow (RDS) gene can cause either retinitis pigmentosa or macular dystrophy. Nat Genet.

[87] Fumagalli A, Ferrari M, Soriani N, Gessi A, Foglieni B, Martina E (2001). Mutational scanning of the ABCR gene with double-gradient denaturing-gradient gel electrophoresis (DG-DGGE) in Italian Stargardt disease patients. Hum Genet.

[88] Briggs CE, Rucinski D, Rosenfeld PJ, Hirose T, Berson EL, Dryja TP (2001). Mutations in ABCR (ABCA4) in patients with Stargardt macular degeneration or cone-rod degeneration. Invest Ophthalmol Vis Sci.

[89] De Castro-Miró M, Pomares E, Lorés-Motta L, Tonda R, Dopazo J, Marfany G (2014). Combined genetic and high-throughput strategies for molecular diagnosis of inherited retinal dystrophies. PLoS One.

[90] Downes SM, Holder GE, Fitzke FW, Payne AM, Warren MJ, Bhattacharya SS (2001). Autosomal dominant cone and cone-rod dystrophy with mutations in the guanylate cyclase activator 1A gene-encoding guanylate cyclase activating protein-1. Arch Ophthalmol.

