# Supplementary material for: Next-generation sequencing applied to a large French cone and cone-rod dystrophy cohort: mutation spectrum and new genotype-phenotype correlation
Source: Orphanet J Rare Dis. 2015 Jun 24;10:85. doi: 10.1186/s13023-015-0300-3 (PMC4566196; doi:10.1186/s13023-015-0300-3)
Supplement: Additional file 1: — Panel of 123 known genes responsible for retinal disease with photoreceptor degeneration. [file 13023_2015_300_MOESM1_ESM.doc]

| **Additional File 1. Panel of 123 known genes responsible for retinal disease with photoreceptor degeneration** *(CCRD genes are highlighted in bold)* | |
| --- | --- |
| *Number* | *Gene name* |
| 1 | ***ABCA4*** |
| 2 | ***ADAM9*** |
| 3 | ***AIPL1*** |
| 4 | *ARL6* |
| 5 | *ASTN2* |
| 6 | *ATXN7* |
| 7 | *BBS1* |
| 8 | *BBS10* |
| 9 | *BBS12* |
| 10 | *BBS2* |
| 11 | *BBS4* |
| 12 | *BBS5* |
| 13 | *BBS7* |
| 14 | *BBS9* |
| 15 | *BEST1* |
| 16 | *C1QTNF5* |
| 17 | *C2orf71* |
| 18 | ***C8orf37*** |
| 19 | *CA4* |
| 20 | *CABP4* |
| 21 | ***CACNA2D4*** |
| 22 | *CC2D2A* |
| 23 | *CDH23* |
| 24 | ***CDHR1*** |
| 25 | *CEP290* |
| 26 | ***CERKL*** |
| 27 | *CHM* |
| 28 | *CLN3* |
| 29 | *CLRN1* |
| 30 | *CNGA1* |
| 31 | *CNGB1* |
| 32 | *CRB1* |
| 33 | ***CRX*** |
| 34 | *CYP4V2* |
| 35 | *DFNB31* |
| 36 | *DHDDS* |
| 37 | *EYS* |
| 38 | *FAM161A* |
| 39 | *FLVCR1* |
| 40 | *FSCN2* |
| 41 | *GNPTG* |
| 42 | *GPR98* |
| 43 | ***GUCA1A*** |
| 44 | *GUCA1B* |
| 45 | ***GUCY2D*** |
| 46 | *IDH3B* |
| 47 | *IMPDH1* |
| 48 | *IMPG2* |
| 49 | *INVS* |
| 50 | *IQCB1* |
| 51 | *KCNJ13* |
| 52 | *KLHL7* |
| 53 | *LCA5* |
| 54 | *LRAT* |
| 55 | *LZTFL1* |
| 56 | *MAK* |
| 57 | *MERTK* |
| 58 | *MFRP* |
| 59 | *MKKS* |
| 60 | *MKS1* |
| 61 | *MYO7A* |
| 62 | *NMNAT1* |
| 63 | *NPHP1* |
| 64 | *NPHP3* |
| 65 | *NPHP4* |
| 66 | *NR2E3* |
| 67 | *NRL* |
| 68 | *OAT* |
| 69 | *OFD1* |
| 70 | *OTX2* |
| 71 | *PAF1* |
| 72 | *PANK2* |
| 73 | *PCDH15* |
| 74 | *PDE6A* |
| 75 | *PDE6B* |
| 76 | ***PDE6C*** |
| 77 | *PDE6G* |
| 78 | *PDZD7* |
| 79 | *PEX1* |
| 80 | *PEX7* |
| 81 | *PHYH* |
| 82 | ***PITPNM3*** |
| 83 | *PRCD* |
| 84 | ***PROM1*** |
| 85 | *PRPF3* |
| 86 | *PRPF31* |
| 87 | *PRPF6* |
| 88 | *PRPF8* |
| 89 | ***PRPH2*** |
| 90 | ***RAX2*** |
| 91 | *RBP3* |
| 92 | *RBP4* |
| 93 | *RD3* |
| 94 | *RDH12* |
| 95 | ***RDH5*** |
| 96 | *RGR* |
| 97 | *RHO* |
| 98 | ***RIMS1*** |
| 99 | *RLBP1* |
| 100 | *ROM1* |
| 101 | *RP1* |
| 102 | *RP2* |
| 103 | *RP9* |
| 104 | *RPE65* |
| 105 | ***RPGR*** |
| 106 | ***RPGRIP1*** |
| 107 | *RPGRIP1L* |
| 108 | *SAG* |
| 109 | ***SEMA4A*** |
| 110 | *SNRNP200* |
| 111 | *SPATA7* |
| 112 | *TMEM237* |
| 113 | *TOPORS* |
| 114 | *TRIM32* |
| 115 | *TTC8* |
| 116 | *TTPA* |
| 117 | *TULP1* |
| 118 | ***UNC119*** |
| 119 | *USH1C* |
| 120 | *USH1G* |
| 121 | *USH2A* |
| 122 | *WDPCP* |
| 123 | *ZNF513* |
