# Supplementary material for: Next-generation sequencing applied to a large French cone and cone-rod dystrophy cohort: mutation spectrum and new genotype-phenotype correlation
Source: Orphanet J Rare Dis. 2015 Jun 24;10:85. doi: 10.1186/s13023-015-0300-3 (PMC4566196; doi:10.1186/s13023-015-0300-3)
Supplement: Additional file 2: — Cosegregation analysis of pathogenic or likely pathogenic mutations. [file 13023_2015_300_MOESM2_ESM.doc]

| **Additional File 2. Cosegregation analysis of pathogenic or likely pathogenic mutations** | | | | | | | | |
| --- | --- | --- | --- | --- | --- | --- | --- | --- |
| **Family** | **ID** | **Family member** | **Clinical status** | **Transmission** | **Gene** | **Exon** | **Allele 1** | **Allele 2** |
| ***Known CD/CRD genes*** | | | | | |  |  |  |
| **107** | **CIC00137** | **I** | Affected | simplex | *ABCA4* | 47 | c.6394G>A p.(E2132K) | c.6394G>A p.(E2132K) |
|  | CIC00138 | F | Unaffected |  |  |  | c.6394G>A p.(E2132K) | - |
|  | CIC02887 | M | Unaffected |  |  |  | c.6394G>A p.(E2132K) | - |
|  | CIC02889 | Si | Unaffected |  |  |  | - | - |
| **120** | **CIC00162** | **I** | Affected | ar | *ABCA4* | 16/31 | c.2463G>A p.(W821*) | het c.4546_4547del p.(Q1516Afs*38) |
|  | CIC00163 | **So** | Unaffected |  |  |  | c.2463G>A p.(W821*) | - |
| **498** | **CIC00765** | **I** | Affected | ar | *ABCA4* | 47 | c.6445C>T p.(R2149*) | c.6445C>T p.(R2149*) |
|  | CIC0786 | Si | Affected |  |  |  | c.6445C>T p.(R2149*) | c.6445C>T p.(R2149*) |
|  | CIC0889 | N | Unaffected |  |  |  | c.6445C>T p.(R2149*) | - |
|  | CIC01117 | Ni | Unaffected |  |  |  | - | - |
| **2132** | **CIC04412** | **I** | affected | simplex | *ABCA4* | 28/34 | c.4234C>T p.(q1412*) | c.4793C>A p.Ala1598Asp |
|  | CIC04413 | M | Unaffected |  |  |  | - | c.4793C>A p.Ala1598Asp |
| **3062** | **CIC05854** | **I** | Affected | ar | *ABCA4* | 35 | c.4919G>A p.(R1640Q) | c.4919G>A p.(R1640Q) |
|  | CIC05855 | F | Unaffected |  |  |  | c.4919G>A p.(R1640Q) | - |
|  | CIC06044 | M | Unaffected |  |  |  | c.4919G>A p.(R1640Q) | - |
|  | CIC06045 | B | Affected |  |  |  | c.4919G>A p.(R1640Q) | c.4919G>A p.(R1640Q) |
|  | CIC06046 | B | Affected |  |  |  | c.4919G>A p.(R1640Q) | c.4919G>A p.(R1640Q) |
| **2506** | **CIC05987** | **I** | Affected | ar | *ABCA4* | 4/22 | c.327dup p.(Q110Sfs*51) | c.3295T>C p.(S1099P) |
|  | CIC04971 | Si | Affected |  |  |  | c.327dup p.(Q110Sfs*51) | c.3295T>C p.(S1099P) |
| **3140** | **CIC05989** | **I** | Affected | simplex | *ABCA4* | 10/34 | c.1302del p.(Q437Rfs*12) | c.4837G>A p.(D1613N)+c.5318C>T p.(A1773V) |
|  | CIC05990 | F | Unaffected |  |  |  | - | c.4837G>A p.(D1613N) +c.5318C>T p.(A1773V) |
| **3255** | **CIC06170** | **I** | Affected | simplex | *ABCA4* | 14/IVS 24+44 | c.2034G>T p.(K678N) + c.6089G>A p.(R2030Q) | c.3607+3A>T r.(spl?) |
|  | CIC06171 | F | Unaffected |  |  |  | - | c.3607+3A>T r.(spl?) |
| **3786** | **CIC06913** | **I** | Affected | ar | *ABCA4* | 21 | c.3056C>T p.(T1019M) | c.3056C>T p.(T1019M) |
|  | CIC06914 | Ni | Unaffected |  |  |  | - | - |
|  |  |  |  |  |  |  |  |  |
|  |  |  |  |  |  |  |  |  |
|  |  |  |  |  |  |  |  |  |
|  |  |  |  |  |  |  |  |  |
|  |  |  |  |  |  |  |  |  |
|  |  |  |  |  |  |  |  |  |
| **605** | **CIC00963** | **I** | Affected | ar | *TULP1* | 11 | c.1087G>A p.(G363R) | c.1087G>A p.(G363R) |
|  | CIC00964 | C | Unaffected |  |  |  | - | - |
|  | CIC07721 | Si | Affected |  |  |  | c.1087G>A p.(G363R) | c.1087G>A p.(G363R) |
| **142** | **CIC00190** | **I** | Affected | simplex | *AIPL1* | 5/5 | c.769C>T p.(L257F) | c.767T>G p.(I256S) |
|  | CIC00191 | M | Unaffected |  |  |  | - | c.767T>G p.(I256S) |
|  | CIC00192 | F | Unaffected |  |  |  | c.769C>T p.(L257F) | - |
| **3342** | **CIC06321** | **I** | Affected | simplex | *RPGRIP1* | 14 | c.2021C>A p.(P674H) | c.2021C>A p.(674H) |
|  | CIC06322 | M | Unaffected |  |  |  | c.2021C>A p.(P674H) | - |
| **718** | **CIC01196** | **I** | Affected | simplex | *PROM1* | 12 | c.1354dup p.(Y452Lfs*13) | c.1354dup p.(Y452Lfs*13) |
|  | CIC01197 | M | Unaffected |  |  |  | c.1354dup p.(Y452Lfs*13) | - |
|  | CIC01198 | F | Unaffected |  |  |  | - | c.1354dup p.(Y452Lfs*13) |
| **2490** | **CIC04945** | **I** | Affected | simplex | *PROM1* | IVS 13/23 | c.1579-1G>C r.(spl?) | c.2383T>C p.(W795R) |
|  | CIC04946 | F | Unaffected |  |  |  | - | c.2383T>C p.(W795R) |
| **3589** | **CIC06642**  CIC06698 | **I**  Si | Affected  Affected | ad | *PROM1* | 1 | c.7dup p.L3Pfs*28  c.7dup p.L3Pfs*28 | -  - |
| **117** | **CIC00535** | **I** | Affected | ad | *PROM1* | 10 | c.1117C>T p.(R373C) | - |
|  | CIC00159 | So | Affected |  |  |  | c.1117C>T p.(R373C) | - |
| **220** | **CIC00324** | **I** | Affected | ad | *GUCY2D* | 13 | c.2512C>T p.(R838C) | - |
|  | CIC00325 | M | Affected |  |  |  | c.2512C>T p.(R838C) |  |
| **1374** | **CIC03249** | **I** | Affected | ad | *GUCY2D* | 13 | c.2512C>T p.(R838C) | - |
|  | CIC03250 | So | Affected |  |  |  | c.2512C>T p.(R838C) | - |
|  | CIC03878 | So | Affected |  |  |  | c.2512C>T p.(R838C) | - |
|  | CIC06912 | D | Affected |  |  |  | c.2512C>T p.(R838C) | - |
| **2088** | **CIC04347** | **I** | Affected | ad | *GUCY2D* | 13 | c.2512C>T p.(R838C) | - |
|  | CIC04348 | So | Affected |  |  |  | c.2512C>T p.(R838C) | - |
| **398** | **CIC00597** | **I** | Affected | simplex | *GUCY2D* | 14 | c.2747T>C p.(I916T) | - |
|  | CIC00598 | Si | Unaffected |  |  |  | - | - |
|  | **CIC08737** | M | Unaffected |  |  |  | - | - |
| **2115** | **CIC06757** | **I** | Affected | ad | *PRPH2* | 1 | c.514C>T p.(R172W) | - |
|  | CIC06756 | Si | Affected |  |  |  | c.514C>T p.(R172W) | - |
|  | CIC06921 | C | Affected |  |  |  | c.514C>T p.(R172W) | - |
|  | CIC04385 | C | Affected |  |  |  | c.514C>T p.(R172W) | - |
| **1581** | **CIC03621** | **I** | Affected | ad | *PRPH2* | 1 | Deletion | - |
|  | CIC03620 | So | Affected |  |  |  | Deletion |  |
| **2502** | **CIC04965** | **I** | Affected | ad | *CRX* | 4 | c.608_609del p.(S203Ffs*32) | - |
|  | CIC05197 | D | Affected |  |  |  | c.608_609del p.(S203Ffs*32) | - |
|  | CIC05198 | So | Affected |  |  |  | c.608_609del p.(S203Ffs*32) | - |
| **1371** | **CIC03241** | **I** | Affected | simplex | *CRX* | 4 | c.564dup p.(A189Rfs*47) | - |
|  | CIC03242 | M | Unaffected |  |  |  | - | - |
|  | CIC03243 | F | Unaffected |  |  |  | - | - |
| **1682** | **CIC03750** | **I** | Affected | simplex | *CRX* | 3 | c.121C>T p.(R41W) | - |
|  | CIC04718 | M | Unaffected |  |  |  | - | - |
|  | CIC04719 | F | Unaffected |  |  |  | - | - |
| **2851** | **CIC05563** | **I** | Affected | ad | *SEMA4A* | 4 | c.302T>C p.(I101T) | - |
|  | CIC05564 | M | Unaffected |  |  |  | - | - |
|  | CIC08686 | F | Affected |  |  |  | c.302T>C p.(I101T) | - |
| ***Other retinal disease genes*** | | | | | |  |  |  |
| **1932** | **CIC01571** | **I** | Affected | ar | *C2Orf71* | 1 | c.2950C>T p.(R984*) | c.2950C>T p.(R984*) |
|  | CIC04118 | M | Unaffected |  |  |  | c.2950C>T p.(R984*) | - |
|  | CIC04119 | F | Unaffected |  |  |  | - | c.2950C>T p.(R984*) |
|  | CIC07775 | C | Affected |  |  |  | c.2950C>T p.(R984*) | c.2950C>T p.(R984*) |
|  | CIC07776 | C | Affected |  |  |  | c.2950C>T p.(R984*) | c.2950C>T p.(R984*) |
|  | CIC07777 | A | Unaffected |  |  |  | c.2950C>T p.(R984*) | - |
| **1819** | **CIC03953** | **I** | Affected | simplex | *EYS* | 11/14 | c.1673G>A p.(W558*) | c.2234A>G p.(N745S) |
|  | CIC03954 | M | Unaffected |  |  |  | - | c.2234A>G p.(N745S) |
|  | CIC04065 | F | Unaffected |  |  |  | c.1673G>A p.(W558*) | - |
| **751** | **CIC01242** | F | Affected | ar | *MERTK* | 3_19 | c.del3_19 | c.del3_19 |
|  | CIC01244 | M | Unaffected |  |  |  | c.del3_19 | - |
|  | CIC01243 | Si | Affected |  |  |  | c.del3_19 | c.del3_19 |
| **3472** | **CIC06499** | **I** | Affected | simplex | *NMNAT1* | 5/5 | c.619C>T p.(R207W) | c.769G>A p.(E257K) |
|  | CIC06500 | M | Unaffected |  |  |  | c.619C>T p.(R207W) | - |
|  | CIC06597 | F | Unaffected |  |  |  | - | c.769G>A p.(E257K) |
| **3191** | **CIC07241** |  | Affected | ar | *RDH12* | 7 | c.464C>T p.(T155I) | c.464C>T p.(T155I) |
|  | CIC07239^*^ | F | Affected |  |  |  | c.464C>T p.(T155I) | c.464C>T p.(T155I) |
|  | CIC07240 | Si | Affected |  |  |  | c.464C>T p.(T155I) | c.464C>T p.(T155I |
| **4119** | **CIC07447** | **I** | Affected | simplex | *RDH12* | 8 | c.806_810del p.(A269Gfs*2) | c.403A>G p.( K135E) |
|  | CIC07448 | M | Unaffected |  |  |  | c.806_810del p.(A269Gfs*2) | - |
| **604** | **CIC00953** | **I** | Affected | simplex | *IQCB1* | 6/8 | c.424_425del p.(F142Pfs*5) | c.686del p.(T229Mtfs*) |
|  | CIC0954 | M | unaffected |  | *IQCB1* |  | c.424_425del p.(F142Pfs*5) | - |
| **2675** | **CIC05272** | **I** | Affected | ad | *BEST1* | 4 | c.76G>A p.(V26M) | - |
|  | CIC05537 | Si | Affected |  |  |  | c.76G>A p.(V26M) | - |
| **831** | **CIC01380** | **I** | Affected | ar | *CRB1* | 11 | c.3994T>G p.(C1332G) | c.3994T>G p.(C1332G) |
|  | CIC04916 | M | Unaffected |  |  |  | c.3994T>G p.(C1332G) | - |
| **2720** | **CIC05379** | **I** | Affected | simplex | *FSCN2* | 1 | c.574C>T p.(R182C) | - |
|  | CIC05381 | So | Unaffected |  |  |  | - | - |
| **1791** | **CIC05007** | **I** | Affected | ad | *ROM1* | 1 | c.339del P(L114Sfs*8) | - |
|  | CIC03914 | Si | Affected |  |  |  | c.339del P(L114Sfs*8) | - |
| **I:index; M: mother; F: father; Si: sister; B: brother; D: daughter; So: son; N: nephew; Ni: niece; C:cousin; A: aunt**  **^*^ CIC07239 is also from a consanguineous union.** | | | | | | | | |
