# Supplementary material for: Next-generation sequencing applied to a large French cone and cone-rod dystrophy cohort: mutation spectrum and new genotype-phenotype correlation
Source: Orphanet J Rare Dis. 2015 Jun 24;10:85. doi: 10.1186/s13023-015-0300-3 (PMC4566196; doi:10.1186/s13023-015-0300-3)
Supplement: Additional file 3: — Summary of 8 patients carrying heterozygous pathogenic or possibly pathogenic mutations in ABCA4, IMPG2 and CDHR1. [file 13023_2015_300_MOESM3_ESM.doc]

| **Additional File 3. Summary of 8 patients carrying only one heterowygous pathogenic or possibly pathogenic mutations in genes underlying arIRDs** | | | | | | | | | | | | | |  |
| --- | --- | --- | --- | --- | --- | --- | --- | --- | --- | --- | --- | --- | --- | --- |
| **ID** | **Type** | **Consanguinity** | **Gene** | **NM** | **Genotype** | **Exon** | **cDNA** | **Protein** | **Conservation** | **Polyphen2** | **Sift** | **Mutation**  **Tester** | **References** |  |
| **CIC00884** | simplex |  | *ABC4A* | NM_000350.2 | Het | 42 | c.5882G>A | p.(G1961E) | Highly | Pd | D | Dc | (Lewis et al. 1999)  (rs1800553) |  |
| **CIC03795** | simplex |  | *ABC4A* | NM_000350.2 | Het | 9 | c.1140T>A | p.(N380K) | Moderately | B | D | Dc | (Webster et al. 2001)  (rs61748549) |  |
| **CIC04379** | simplex |  | *ABC4A* | NM_000350.2 | Het | 16 | c.2546T>C | p.(V849A) | Weakly | B | D | Poly | (Webster et al. 2001)  (rs61749435) |  |
| **CIC05758** | simplex | + | *ABC4A* | NM_000350.2 | Het | 18 | c.2690C>T | p.(T897I) | Not | B | D | Dc | (Webster et al. 2001)  (rs61749440) |  |
| **CIC06411** | simplex |  | *ABC4A* | NM_000350.2 | Het | 42 | c.5882G>A | p.(G1961E) | Highly | Pd | D | Dc | (Lewis et al. 1999)  (rs1800553) |  |
| **CIC07267** | simplex |  | *ABC4A* | NM_000350.2 | Het | 18 | c.2690C>T | p.(T897I) | Not | B | D | Dc | (Webster et al. 2001) |  |
| **CIC00680** | simplex |  | *IMPG2* | NM_016247.3 | Het | 3 | c.379G>A | p.R127* | - | - | - | - | (Neveling et al. 2012) |  |
| **CIC07507** | Ar | *+* | *CDHR1* | NM_033100.3 | Het | 16 | c.1863dup | p.(I622Hfs*54) | - | - | - | - | Novel |  |

**Ar: autosomal recessive; Het: heterozygous, Poly: Polymorphism; D: deleterious, Dc: Disease causing**
